# Bioinformatical Analysis of the Sequences, Structures and Functions of Fungal Polyketide Synthase Product Template Domains

**DOI:** 10.1038/srep10463

**Published:** 2015-05-21

**Authors:** Lu Liu, Zheng Zhang, Chang-Lun Shao, Jin-Lan Wang, Hong Bai, Chang-Yun Wang

**Affiliations:** 1 Key Laboratory of Marine Drugs, the Ministry of Education of China, School of Medicine and Pharmacy, Ocean University of China, Qingdao 266003, China; 2 State Key Laboratory of Microbial Technology, Shandong University, Jinan 250100, China; 3 School of Life Science, Shandong University, Jinan 250100, China; 4 Institute of Evolution & Marine Biodiversity, Ocean University of China, Qingdao 266003, China

## Abstract

The product template (PT) domains, specifically in fungal non-reducing polyketide synthases (NR-PKSs), mediate the regioselective cyclization of polyketides dominating the final structures. However, up to date, the systematic knowledge about PT domains has been insufficient. In present study, the relationships between sequences, structures and functions of the PT domains were analyzed with 661 NR-PKS sequences. Based on the phylogenetic analysis, the PT domains were classified into prominent eight groups (I–VIII) corresponding with the representative compounds and cyclization regioselectivity (C2-C7, C4-C9, and C6-C11). Most of the cavity lining residue (CLR) sites in all groups were common, while the regional CLR site mutations resulted in the appearance of finger-like regions with different orientation. The cavity volumes and shapes, even the catalytic dyad positions of PT domains in different groups were corresponding with characteristic cyclization regioselectivity and compound sizes. The conservative residues in PT sequences were responsible for the cyclization functions and the evolution of the key residues resulted in the differentiations of cyclization functions. The above findings may help to better understand the cyclization mechanisms of PT domains and even predict the structural types of the aromatic polyketide products.

One hallmark of fungi is their capacity to synthesize diverse biological polyketide natural products with structural variation during synthesis by polyketide synthase (PKSs)^1^,^2^,^3^. With a few exceptions, a majority of fungal PKSs are iterative type I PKSs^1^,^4^. Fungal PKSs are intrinsically more difficult to be studied than the dissociated bacterial PKSs due to their large sizes (in excess of 200 kDa) and the difficulties with genetic manipulation^5^. In the past decade, more knowledge about biosynthesis mechanisms of fungal polyketide synthases has been acquired. It is generally recognized that most biosynthetic gene clusters including PKSs are silent or expressed at very low levels according to global transcriptomic profile^6^. Fungi have the potential to produce a far greater number of polyketides than the known polyketides isolated from fungi up to date. Therefore, it is essential to investigate and characterize fungal PKSs for enzymatic mechanism elucidation and genetic manipulation to obtain more new metabolites.

The fungal PKSs can be divided into three major classes according to the function and phylogeny, i.e., the non-reducing (NR), the highly-reducing (HR), and the partial-reducing (PR) PKSs^1^. The NR-PKSs synthesize aromatic polyketides, such as carcinogenic mycotoxin aflatoxin^7^. Besides the basic PKS domains, additional functional domains including the starter unit-ACP transacylase (SAT) domain, product template (PT) domain, and thioesterase (TE) releasing domain are unique to the NR-PKSs^1^,^7^,^8^. Recently, the PT domains have been demonstrated to be embedded in controlling specific aldol cyclization and aromatization of the polyketide precursors^7^. There are three commonly cyclizing patterns (C2-C7, C4-C9, and C6-C11), when the first cyclization occurs^9^.

According to an earlier phylogenetic analysis of β-ketoacyl synthase (KS) domains, NR-PKSs were divided into three basal subclades (subclades I–III), each of them characterized a typical domain architecture^10^. Subclade III was characterized by a methyltransferase (CMeT) domain located after the acyl carrier protein (ACP) domain. And subclades I and II diverged after losing the CMeT domain. Later, based on a phylogenetic analysis of PT domains in subclades I and II from known functional NR-PKSs, the PT domains were classified into five groups (groups I–V) corresponding to the cyclization regioselectivity at C2-C7, C4-C9, and C6-C11 as well as compound sizes^9^. Recently, the PT domains with associated metabolites in subclade III were further classified into two groups (groups VI and VII) responsible to produce aromatics with C2-C7 cyclization^11^.

Up to date, only one PT domain (group IV) crystal structure has been studied from *Aspergillus parasiticus* PksA, which participates in aflatoxin B1 biosynthesis^12^. The PT structure of PksA displays a distinct ‘double hot dog’ (DHD) fold and the two DHD monomers associate *via* a PT-specific sequence insertion. The residues important for substrate binding and catalysis were revealed, and an internal pocket was identified, which can be visually divided into three regions, phosphopantetheine (PPT)-binding region, cyclization chamber, and hydrophobic hexyl-binding region. The crystal structure of PT domain in PksA provided a vital model for the structural study on other PT domains of which structures were still unknown.

So far, the systematic knowledge about PT domains has been insufficient. The relationships between sequences, structures and functions of the PT domains have been unclear. In this study, the NR-PKS phylogenetic tree and PT phylogenetic tree were established based on all known fungal NR-PKSs protein sequences. The three-dimensional structures of different PT domains were modeled, and the relationships between catalytic pocket shapes and sizes, regioselective cyclization, and compound sizes were analyzed. The influence of the PT domain sequence variations on the differentiations of structures and functions was also discussed.

## Results and Discussion

### Phylogenetic analysis of fungal NR-PKSs

All of the known fungal NR-PKSs amino acid sequences were searched and screened from the NCBI database to establish phylogenetic trees (Supplementary Table S1). The collected NR-PKSs dataset comprised of 661 sequences, of which 627 sequences were derived from 187 strains of ascomycetes and 34 sequences from 29 strains of basidiomycetes.

**Figure 1:**
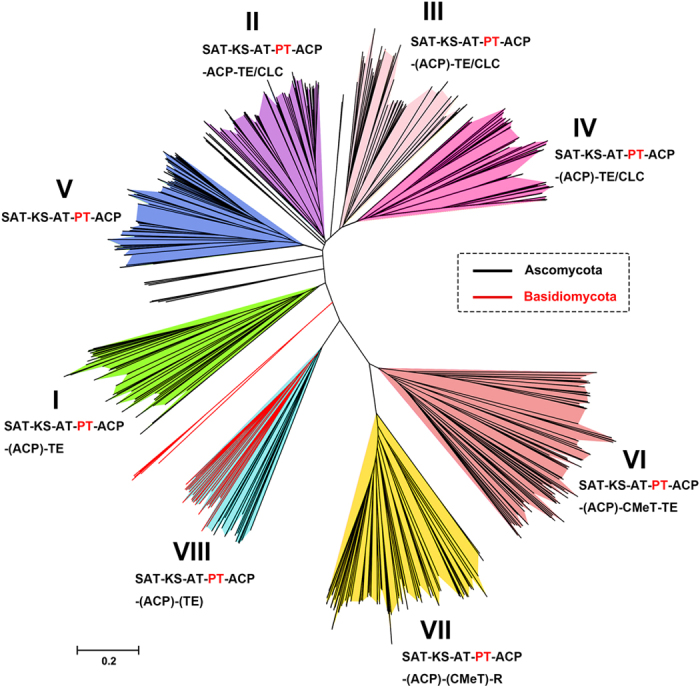
NR-PKS phylogenetic tree of 661 sequences from ascomycetes and basidiomycetes. **** The branches of eight groups have been colored, each of which shares a common organization of domains (those in parentheses are variable in their presence/absence within that group). Domain abbreviations: SAT = starter unit-ACP transacylase, KS = ketosynthase, AT = acyl transferase, PT = product template, ACP = acyl carrier protein, TE = thioesterase, TE/CLC = thioesterase/Claisen cyclase, CMeT = C-methyltransferase, R = reductase. The tree is drawn to scale, with branch lengths in the same units as those of the evolutionary distances used to infer the phylogenetic tree.

The NR-PKS phylogenetic tree was constructed on the basis of the phylogeny and domain architectures. The resulting NR-PKS phylogenetic tree clearly classified the collected NR-PKS sequences into eight major groups (groups I–VIII) except a few sequences (Fig. 1). Compared with the previous report^11^, besides the seven groups, a new group (VIII) appeared in this tree obviously different from the other groups. The NR-PKSs from ascomycetes were found to cover throughout all eight groups, however the NR-PKSs from basidiomycetes only appeared in group VIII and rare intermediate clades. It was found that the NR-PKSs from each genus fell into no more than six groups. The NR-PKSs from genera *Aspergillus*, *Bipolaris*, *Colletotrichum* and *Talaromyces* were found in six groups in the phylogenetic tree.

All of the NR-PKSs contain SAT, KS, AT, PT and ACP domains with the PT domains locating between AT and ACP domains. Moderate differences of domain architectures were observed in eight groups. The C-terminus TE/CLC domains responsible for releasing products in all members in group V were absent. Most members in groups VI and VII were characterized by a CMeT domain located after the ACP domain, which can produce aromatics with methyl branching on their benzene rings. Most NR-PKSs (groups I–IV, VI, VIII) showed an additional ACP domain. A previous study revealed that the product-releasing domains including TE/CLC in each group could be further distinguished by different functions^13^. It suggests that more information about NR-PKS sequences should be accumulated to clarify the phylogenetic relationships between different groups in the future.

### Differentiation of PT domain functions

The phylogenetic tree of PT domains from 661 NR-PKS sequences was also constructed. The PT sequence boundaries were defined by computation tools according to the NR-PKSs differentiation and the sequence length is about 300 ~ 400 amino acids. The established PT domain phylogenetic tree was similar to that of NR-PKSs, also classified into eight groups (Supplementary Fig. S1).

**Figure 2:**
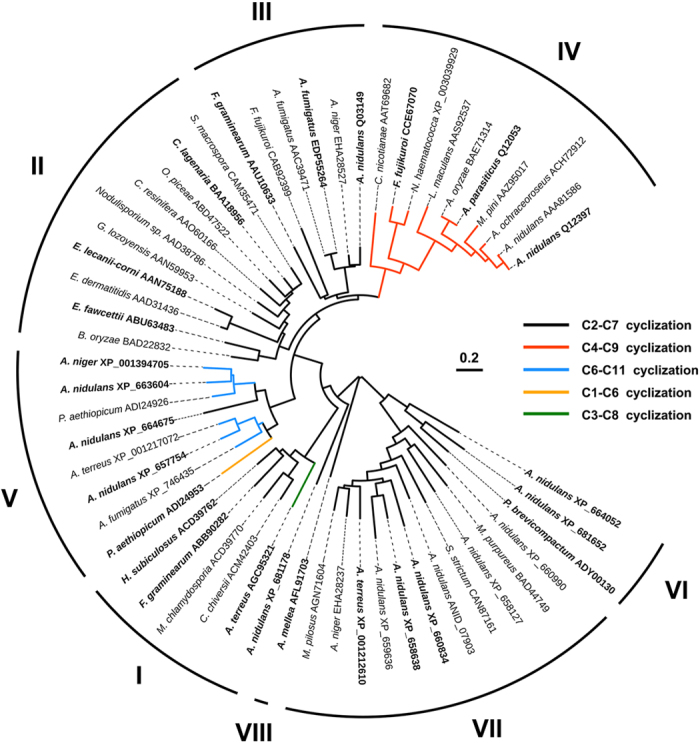
Phylogenetic tree of PT domains of selected 55 NR-PKSs. **** PT domains of 55 NR-PKSs are related to cyclization regioselectivities. Phylogenetic analysis was conducted using the bootstrap neighbor joining method. Bold branches indicate sequences which have been used in structure modeling. The tree is drawn to scale, with branch lengths in the same units as those of the evolutionary distances used to infer the phylogenetic tree.

Until now, 55 NR-PKSs among the 661 NR-PKS sequences have been investigated for their regioselective cyclization modes (Supplementary Table S2). Successively, the PT domain phylogenetic tree from these 55 NR-PKSs was established based on their regioselective cyclization modes (Fig. 2). These 55 PT domains were also found to cover all eight groups (groups I–VIII), responding to the common first-ring cyclization of C2-C7 (groups I–III, VI–VIII), C4-C9 (group IV) and C6-C11 (group V), and the rare cyclization of C1-C6 (group V) and C3-C8 (group I).

The phylogenetic analysis suggested that each group of PT domains associates with specific regioselective cyclization and compound size. In group I, most PT domains catalyze the first-ring cyclization *via* C2-C7 aldol condensation. The main NR-PKSs in this group are involved in the biosynthesis of aromatic portions in the resorcylic acid lactones, exemplified by hypothemycin Hpm3^14^, radicicol RDC1^14^/RADS2^15^, and zearalenone PKS13^16^. Group I also includes a single aromatic ring synthase, the orsellinic acid OrsA from *Aspergillus nidulans*^17^. As an exception, the dehydrocurvularin AtCURS2 from *Aspergillus terreus* forms the first rings *via* C3-C8 rather than C2-C7^18^.

In group II, the NR-PKSs include the most known THN synthases from a variety of genera responsible for the synthesis of 1,3,6,8-tetrahydroxynaphthalene (THN) analogues^19^. The PT domains of this group catalyze the aldol condensation *via* C2-C7 with pentaketide backbones. The TE/CLC domains in this group are in charge of cyclizing the second rings.

The NR-PKSs in group III synthesize longer polyketide chains *via* C2-C7 cyclization, such as naphthopyrone heptaketide synthase Alb1^20^ and bikaverin nonaketide synthase PKS4^21^. Interestingly, the WA synthase from *A. nidulans* also generates pentaketide THN with shorter polyketide chains^11^. The previously biochemical analysis showed that THN is a result of shortening of the heptaketide YWA1, a common metabolite in group III synthesized by WA synthase 11,22.

All the PT domains in group IV cyclize the first ring *via* C4-C9 regiospecificity. The only one crystal structure of PT domain in PksA from *A. parasiticus*^12^ is classified into group IV. The NR-PKSs of this group contain the analogues of decaketide synthase PksA, such as aflatoxin/sterigmatocystin synthases^23^,^24^. The NR-PKSs in this group also include heptaketide synthases such as cercosporin CTB1^25^ and naphthaldehyde PKS1^26^.

All of the NR-PKSs in group V lack TE/CLC domains and they mainly form the first ring *via* C6-C11 register. It seems that these NR-PKS members produce multiple aromatic polyketides with the most different polyketide backbones by regioselectivity cyclization modes. Group V is composed of the NR-PKSs catalyzing the synthesis of different polyketide backbones, such as griseofulvin heptaketide synthase GsfA from *Penicillium aethiopicum*^27^, emodin octaketide synthase ACAS from *A. terreus*^28^, asperthecin nonaketide synthase AptA from *A. nidulans*^29^ and TAN-1612 decaketide synthase AdaA from *Aspergillus. niger*^30^. It should be noted that heptaketide synthase GsfA^27^ catalyze the unorthodox C1-C6 aldol cyclization. Interestingly, heptaketide synthase PkgA^11^ for C2-C7 aldol cyclization was also found in this group.

All of the NR-PKSs in groups VI and VII associate with C2-C7 specific cyclization, and most of them have a CMeT domain. The compounds derived from group VI are single aromatic ring natural products such as 5-methylorsellinic acid from *Penicillium brevicompactum*^31^. While the metabolites synthesized by the NR-PKSs of group VII were found to have different polyketide backbones, such as tetraketide orsellinaldehyde^11^, pentaketide citrinin^32^, hexaketide rubropunctatin^33^, octaketide 2,4-dihydroxy-6-[(3E,5E,7E)-2-oxonona-3,5,7-trienyl]benzaldehyde^11^, and nonaketide 2,4-dihydroxy-3-methyl-6-(2-oxopropyl)benzaldehyde^11^.

Group VIII is a newly separate group according to our phylogenetic analysis. Different from other groups, this group contains not only the NR-PKS members from ascomycetes and but also from basidiomycetes. The only studied polyketide synthase in group VIII is orsellinic acid synthase ArmB from *Armillaria mellea* with C2-C7 cyclization^34^.

The above phylogenetic analysis for PT domains indicated that the cyclization modes of C2-C7, C4-C9, C6-C11, C1-C6, and C3-C8 were found in eight groups. C2-C7 cyclization is the most common mode that spreads over seven groups except group IV. And the C4-C9 and C6-C11 cyclization modes only appear in groups IV and V, respectively.

### Structural analysis of PT domains

**Figure 3:**
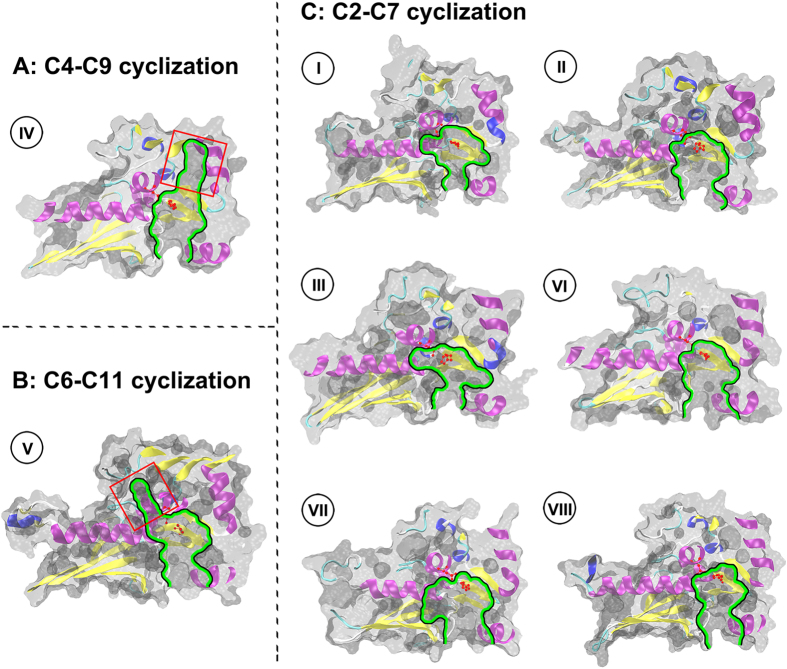
Comparison of pocket shapes among eight groups of PT domains. **** A. The PT structure for C4-C9 cyclization is exemplified by 3HRR PDB structure from group IV. B. The PT structure for C6-C11 cyclization is exemplified by XP_657754 structural model from group V. C. The PT structure for C2-C7 cyclization is exemplified by XP_681178 from group I, BAA18956 from group II, Q03149 from group III, XP_681652 from group VI, XP_658638 from group VII and AFL91703 from group VIII. All PT structures for C2-C7 cyclization are structural models. Surface representations of the pocket in the PT monomer are shown using green outline. The special finger-like regions of group IV and group V are respectively indicated with red boxes. The side chain atoms of catalytic dyads (His/Asp) in all groups are highlighted.

In order to describe the PT domain structures corresponding to various cyclization modes, the catalytic pockets of different PT domains were analyzed and compared. The PT domain of *A. parasiticus* PksA responsible for C4-C9 cyclization has been the only PT crystal structure reported to date. The internal pocket of this PT domain extends 30 Å from the surface to the bottom (Fig. 3A). The catalytic pocket is divided into three regions: the phosphopantetheine (PPT)-binding region, the cyclization chamber, and the hydrophobic hexyl-binding region. The proposed catalytic dyad (Asp 1543/His 1345) locates at the middle of the pocket, i.e. the cyclization chamber, and initiates the regiospecific cyclizations appropriately.

The three-dimensional PT structures of eight groups were built by comparative protein modeling methods. For each group except for group VIII, at least three representative PT sequences were used to build three-dimensional structures (Supplementary Table S3). All of the sequences analyzed by comparative protein modeling methods were highlighted in bold in PT domain phylogenetic tree (Fig. 2). In order to make the simulated structures more convincing, three structural models for each PT domain were acquired by SWISS-MODEL^35^, I-TASSER^36^, and PHYRE2^37^, respectively. All the structural models were refined in the atomic-level by the fragment-guided molecular dynamics (FG-MD) simulations^38^. According to quality assessment by TM-score^39^ and Ramachandran plot^40^, the accuracy of all the PT protein structural models were acceptable.

Based on the above simulated structures, the PT structures for C6-C11 cyclization were found to have a catalytic pocket deep about 30 Å from the surface to the bottom. Furthermore, the proposed catalytic dyad locates in the middle of the pocket. These characteristics of C6-C11 cyclization are similar to that of C4-C9 register (Fig. 3B). The obvious difference between C6-C11 and C4-C9 was found in the finger-like regions. Contrasted to the long, straight region of C4-C9, the finger-like region of C6-C11 orientates at a specific angle. In the PT structure for C2-C7, contrasting to that of C4-C9 and C6-C11, the depth of catalytic pockets are obviously shallow, range from 15 Å to 20 Å. It should be pointed out that C2-C7 catalytic pockets in groups I–III and VI–VIII are lack of the finger-like regions and the catalytic dyads locate at the bottom of the pockets (Fig. 3C).

The above results indicated that the features of PT pockets adapt with various catalytic mechanisms. In C4-C9 and C6-C11 cyclization modes, the catalytic pockets have larger sizes with the finger-like regions. The previous crystal structure study revealed that the hydrophobic hexyl-binding region (the finger-like region) of PT domain in aflatoxin synthase PksA can perfectly adapt to accept the substrate hexanoyl starter unit^12^. In present study, based on the three-dimensional model analysis, the PT domains of C4-C9 cyclization present the similar finger-like regions and generate aflatoxin analogues (Supplementary Table S2). While in the PT domains of C6-C11 cyclization, the finger-like regions associated with diverse polyketides was observed for the first time. It suggests that the finger-like regions for C4-C9 and C6-C11 cyclizations can accommodate the relevant chains of these compounds during the ring formation processes. Correspondingly, in the PT crystal structure of PksA, the proposed catalytic dyad appropriately locates in the middle of catalytic pockets^12^. Similarly, in the PT domains of C4-C9 and C6-C11, the proposed catalytic dyads also locate in the middle of catalytic pockets like PksA to initiate the regiospecific cyclizations. In all groups of C2-C7 PT catalytic pockets, the finger-regions have not been observed and the catalytic dyads locate at the bottom of the pockets. It suggests that these catalytic dyad positions are corresponding with the C2-C7 cyclization and the polyketide chains could extend or curve to close to the catalytic dyads during the ring cyclization processes.

**Figure 4:**
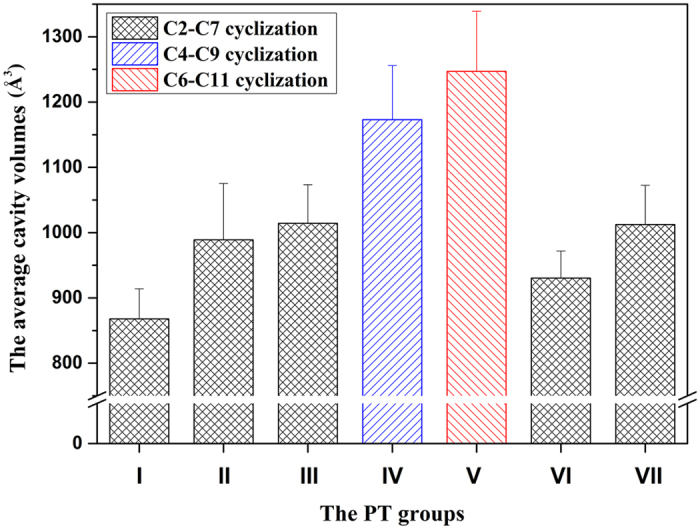
The average value of cavity volumes for PT groups (groups I–VII). **** The cavity volumes were calculated and identified by CASTp with 1.4 Angstroms probe radius.

In order to analyze the relationship between the cavity volumes of PT pockets and compound sizes, the cavity volumes of PT catalytic pockets were calculated by using CASTp (Supplementary Table S4). It was found that the average cavity volume of C6-C11 (group V) pockets is the biggest one, followed by the C4-C9 (group IV) pockets (Fig. 4). The cavity volumes of C2-C7 pockets in five groups (groups I–III, VI, and VII) are significantly smaller than those of C6-C11 and C4-C9 pockets, with groups I, II and VI smaller than those of groups III and VII.

The C2-C7 cyclization modes cover the groups I–III, and VI–VIII. The NR-PKSs in groups I, VI and VIII generate the tetraketides as the common metabolites. Interestingly, the compounds generated by groups I, II and VI, such as orsellinic acid^17^,^31^ and THN^19^, are smaller than the compounds derived from groups III and VII, such as YWA1^11^ and asperfuranone^41^. It seems that the slightly bigger volumes of PT catalytic pockets in groups III and VII make it possible for the longer chains to be accommodated and cyclized. In fact, the THN synthases appear in both groups II and III^11^,^19^, and most are involved in group II. As mentioned above, in group III, the THN is derived from heptaketide YWA1 synthesized by WA synthase^22^. The NR-PKSs in group III usually synthesize compounds with longer chains and multiply fused-rings, and most of them are heptaketides (Supplementary Table S2). In this study, the phylogenetic analysis showed that groups II and III have a near homology relationship in evolutionary process, and the structural analysis indicated that the pocket volumes of PT domains of group III are bigger than those of group II. Similarly, group IV (C4-C9) has a closer homology relationship with group III than with group II, and the catalytic pockets of group IV possess larger cavity volumes than those of group III. Furthermore, the compounds synthesized by group IV have longer chains, such as the main metabolite, decaketide aflatoxin^23^,^42^,^43^ (Supplementary Table S2).

In addition to the three common cyclizations, the rare cyclizations of C1-C6 and C3-C8 were also analyzed (Supplementary Figs. S2 & S3). Currently, there is only one case of C1-C6 aldol condensation in group V which catalyze the heptaketide cyclization. Structure modeling showed that the cavity volume of C1-C6 is merely about one half of the C6-C11 volume. The PT pocket of C1-C6 cyclization also lacks finger-like region and the catalytic dyad locates at the bottom of the pocket. So far, only two heptaketide synthases *P. aethiopicum* GsfA and *A. nidulans* PkgA in group V have been found to catalyze C1-C6 and C2-C7 heptaketide cyclizations, respectively. It is speculated that the cavity structures of group V cannot be suitable for the C6-C11 heptaketide cyclization, leading to the emergence of special C1-C6 and C2-C7 heptaketide cyclizations (Supplementary Table S2). The cavity shape of the sole case of C3-C8 cyclization is similar to that of C2-C7 in group I. However, due to its residue mutations, the cavity volume of C3-C8 is relatively small.

The TE/CLC domains in group V are absent reflected by the phylogenetic analysis of NR-PKSs, while these NR-PKSs still have the ability to generate multi-ring compounds (Supplementary Table S2). The previous research on crystal structure supported that the PT catalytic pocket of PksA in group IV can accommodate the regiospecific cyclizations for two rings^12^. According to the above analysis, the PT domains with the finger-like regions in group V have the larger cavity volumes, which may enable the PT domains to catalyze more cyclization processes.

### Sequence differentiation of PT domains

The cavity lining residue (CLR) sites of each PT sequence were identified from the catalytic pockets selected by CASTp^44^, and then the CLR sites of each group were determined by multiple sequence alignment. The CLR sites of each group were considered only if they presented in the catalytic pockets at least twice in three proteins of each PT group. The CLR sites of seven groups (groups I–VII) were identified, except for group VIII with only one known regioselective cyclization sequence.

**Figure 5:**
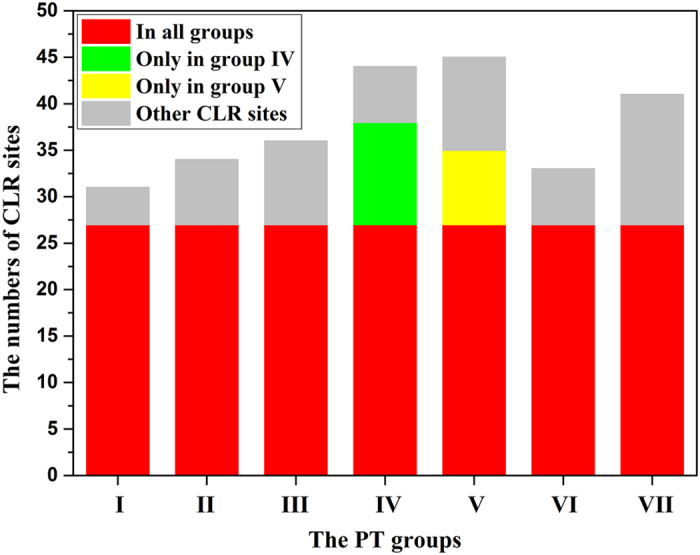
Statistical analysis of CLR sites of PT groups (groups I–VII). **** The red block, 27 common CLR sites in groups I–VII; the green block, 11 specific CLR sites in group IV; the yellow block, 8 specific CLR sites in group V; the gray block, the total CLR sites except for the common CLR sites and the specific CLR sites in groups IV and V.

It was indicated that the catalytic pockets of each PT group are composed of 31–45 CLR sites (Table 1). The CLR site number and cavity volume of each PT group showed a positive correlation (r = 0.93, p < 0.01). Groups IV and V have the most CLR sites corresponding with the biggest cavity volumes. A total of 64 CLR sites appear in seven PT groups, including the CLR sites only in one group (Supplementary Table S5). Multiple sequence alignment revealed that among the predicted CLR sites, 27 CLR sites are common in seven groups (group I–VII), accounting for 87% of total CLR sites in group I (the most), and 60% of total CLR sites in group V (the minimum) (Fig. 5). These results suggested that most of the CLR sites are consistent in different groups, despite the various shapes and sizes of catalytic pockets.

The sequence differentiation of PT domains were also analyzed. The conservation analyses of all amino acid residues were performed with the evolutionary conservation scores calculated by ConSurf^45^. The evolutionary conservation of amino acid residues could be estimated with ConSurf based on the phylogenetic relations of homologous sequences. The analysis results showed that the proposed catalytic dyads (Asp 1543/His 1345 as in *A. parasiticus* PksA) are highly conserved.

**Figure 6:**
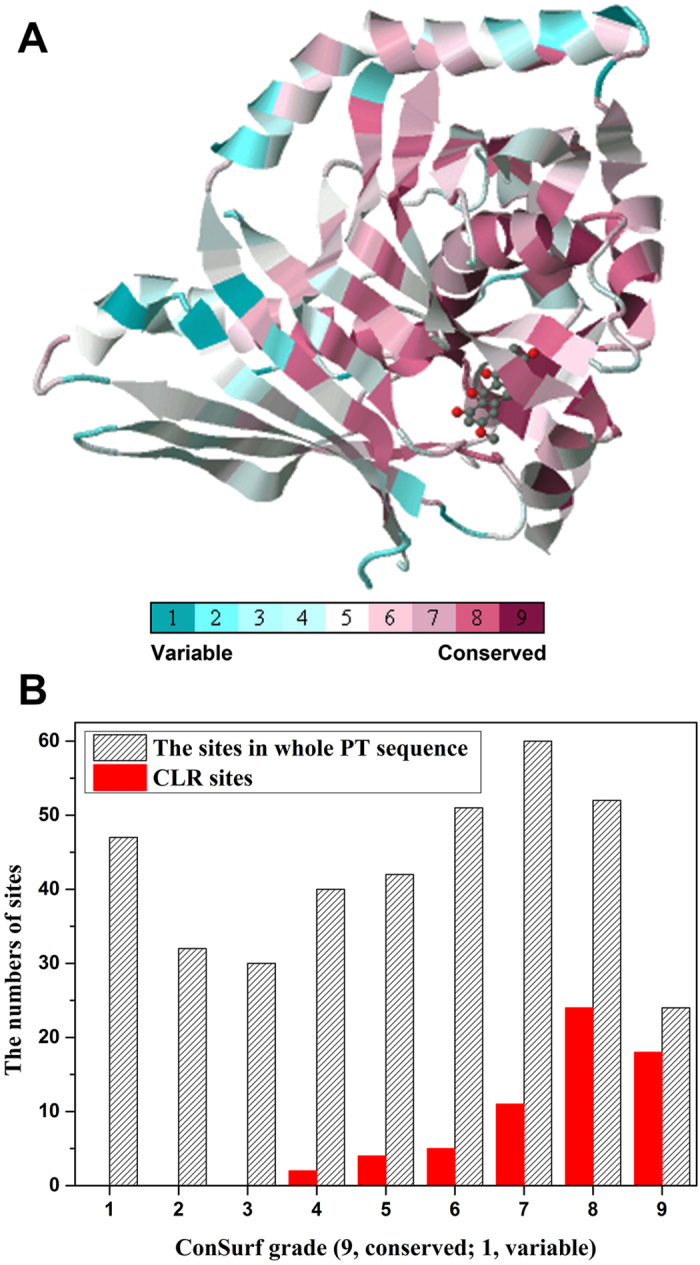
Conservation scale analysis of residue sites in PT domains. **** A. The structure of 3HRR HC8-bound structure belonging to Q12053. Conservation scale is defined from the most variable residue sites (grade 1, color represented by turquoise) which are considered as rapidly evolving to conservative residue sites (grade 9, color represented by maroon) which are considered as slowly evolving. B. The statistical analysis of PT sequence sites and CLR sites with ConSurf grades. The ConSurf grades were calculated with 661 PT sequences. The whole PT sequence was exemplified by Q12053 with 378 sites. The CLR sites were 64 in total from groups I–VII, including the CLR sites appearing only in one group.

Furthermore, most of the conserved residues in 661 PT sequences locate at the catalytic pockets (Fig. 6A, maroon residues). Based on the statistical analysis, the conservation of residues in the whole sequences is low (~35%, ConSurf grade 7–9), while for all of the CLR sites (a total of 64 sites, including the CLR sites appearing only in one group), the proportion of conserved residues reaches 83% (Fig. 6B). There are 25 residues (ConSurf grade 7–9) in the 27 common CLR sites are conserved, accounting for more than 92%. Particularly, there are 15 residues are the most conserved residues (ConSurf grade 9) in the common CLR sites. Therefore, the residues in CLR sites are relatively conserved in evolutionary process comparing with the residues in the whole PT sequences. These results suggested that the cyclization functions significantly constrain the evolution of PT sequences. In a previous study, the 6 residues in group IV, H1345, D1543, Q1547, N1554, T1546, and N1568 (number to *A. parasiticus* PksA prevail) belonging to the 27 common CLR sites have been validated to be important residues for activity by mutations^12^.

In groups IV and V, however, besides the 27 common CLR sites, 11 specific CLR sites and 8 specific CLR sites were found to only appear in the catalytic pockets of these two groups, respectively (Supplementary Table S5). These residue sites may associate with the finger-like regions of internal pockets of these two groups. The CLRs in group IV, G1491, M1495, M1498 and A1499 (number to *A. parasiticus* PksA prevail), are involved in the formation of finger-like region. In the corresponding positions of other groups, these CLRs are replaced by the bulkier residues (e.g., Val, Phe, and Leu), preventing the formation of finger-like region. The previous experimental data showed that the specific CLR G1491 was mutated to Leu, resulting in no apparent activity of the PT domain^12^. Additionally, in the catalytic pockets of group IV, the CLRs A1397 and L1622 replace the conserved Pro and Phe in other groups, respectively, resulting in the enlargement of pocket volumes. In group V, V1567 and G1638 (number to *A. nidulans* MdpG prevail) substitute the bulkier residues in other groups, including Phe and Ala, to generate finger-like region. Similarily, M1378 and G1423 in group V take the place of the conserved Val and Pro in other groups, respectively, resulting in the increase of the pocket volumes.

Furthermore, nearby the catalytic dyad, Thr (1354) in group IV and Thr (1385) in group V, are replaced by Pro in all C2-C7 groups. The spatial structure of Pro may influence the ring architecture and prevent the transfering of the first ring with C2-C7 regio-specific cyclization.

The above analysis suggested that the CLRs related with the catalysis are highly conserved in the main catalytic cavity of the pockets. The volume diversification of PT pockets affects the different cyclization regiospecificity and even compound formations. Residue mutation is the main reason to change the sizes and shapes of the PT pockets.

## Conclusions

In present study, the relationships between sequences, structures and functions of PT domains in fungal polyketide synthases were analyzed and elucidated. In the evolutionary process, the PT domains diverge into eight groups with functional distribution to synthesize diverse polyketides with variety of substrates by different cyclization modes. The functional differentiation is attributed to the changes of the cavity shapes and sizes with CLR mutations arised from the sequence variations of PT domains. The above findings may help to better understand the cyclization mechanisms of PT domains and even predict the structural types of the products especially with aromatic polyketide backbones from NR-PKS protein sequences. It could be prospected to manipulate the fungal PKS genes and further regulate the biosynthetic pathway to obtain the target metabolites.

## Methods

### Dataset

The amino acid sequences of 55 fungal NR-PKSs with known cyclization modes were collected from NCBI database (Genbank)^46^ with accession numbers which were obtained from the literature. The homologous sequences of 661 fungal NR-PKSs were searched and obtained by BLAST based on the 55 sequences^47^. The repetitive sequences and partial sequences were eliminated. The accession numbers and related information of NR-PKSs were provided in Supplementary Table S1 and Table S2. The PT domain sequences of NR-PKSs were extracted and calibrated with UMA procedure^48^, SMART^49^, and CDD^50^.

### Phylogenetic Analysis

The NR-PKS sequences and PT sequences were aligned with MAFFT^51^, respectively. Phylogenetic analyses were conducted using MEGA version 6 by the bootstrap neighbor joining method^52^. The evolutionary distances were computed using the Poisson correction method and were in the units of the number of amino acid substitutions per site. The phylogenetic tree was displayed by iTOL^53^.

### Structure modeling

The three-dimensional models of PT domains were constructed using comparative protein modeling methods (Supplementary Table S3). The structural templates for PT domains were identified by BLAST search against PDB or by using threading approach. Three structural models for each PT domain were acquired by SWISS-MODEL^35^, I-TASSER^36^ and PHYRE2^37^, respectively. All the structural models were refined in the atomic-level by the fragment-guided molecular dynamics (FG-MD) simulations^38^. Two forms of quality assessment, TM-score^39^ and Ramachandran plot^40^, have been used to quantitatively assess the accuracy of protein structure predictions.

### Cavity volume and CLR site analysis

The structural mapping and pocket architecture visualization were displayed using VMD^54^. The cavity volumes and the cavity lining residue (CLR) sites were calculated and identified by CASTp with 1.4 Angstroms probe radius^44^, respectively. The cavity volume of each PT protein was defined as the average value of the three models acquired by SWISS-MODEL+MD, I-TASSER+MD, and PHYRE2+MD. The CLR sites of each PT protein were defined as the sites that are present in the catalytic pockets at least twice in three models.

### Evolutional conservation analysis

The evolutionary conservation of amino acid positions in the PT sequences was estimated by using ConSurf algorithm^45^ (Supplementary Table S5). The LG substitution matrix and computation were based on the empirical Bayesian paradigm. Conservation scale was defined from the most variable amino acid positions (grade 1, color represented by turquoise) which were considered as rapidly evolving to conservative positions (grade 9, color represented by maroon) which were considered as slowly evolving.

## Additional Information

**How to cite this article**: Liu, L. *et al.* Bioinformatical Analysis of the Sequences, Structures and Functions of Fungal Polyketide Synthase Product Template Domains. *Sci. Rep.* **5**, 10463; doi: 10.1038/srep10463 (2015).

## Supplementary Material

Supporting Information Supplementary Figures 1-6

## Figures and Tables

**Table 1:** The data of PT domain catalytic pockets.

**Group**^a^	**Regioselectivity cyclization**	**Accession number**	**Number of CLRs**^b^
I	C2-C7	XP_681178, ABB90282, ACD39762	31
II	C2-C7	BAA18956, AAN75188, ABU63483	34
III	C2-C7	Q03149, AAU10633, EDP55264	36
IV	C4-C9	Q12053 (3HRR), Q12397, CCE67070	44
V	C6-C11	XP_657754, XP_663604, XP_001394705	45
VI	C2-C7	XP_681652, ADY00130, XP_664052	33
VII	C2-C7	XP_658638, XP_660834, XP_001212610	41

^a^Group VIII is not considered because only one sequence is studied with regioselective cyclization.

^b^CLRs are present in the catalytic pockets at least twice in three proteins of each PT group.
