# Supplementary material for: Bioinformatical Analysis of the Sequences, Structures and Functions of Fungal Polyketide Synthase Product Template Domains
Source: Sci Rep. 2015 May 21;5:10463. doi: 10.1038/srep10463 (PMC5386248; doi:10.1038/srep10463)
Supplement: Supporting Information — Supplementary Figures 1-6 [file srep10463-s1.pdf]

Bioinformatical Analysis of the Sequences, Structures and Functions of Fungal Polyketide Synthase Product Template Domains

Lu Liu[1], Zheng Zhang[2], Chang-Lun Shao[1], Jin-Lan Wang[3], Hong Bai[1] & Chang-Yun Wang[1,4]

Supplementary Materials

Supplementary Fig. S1

Phylogenetic analysis of 661 PT domains..**page 1**

Supplementary Fig. S2

The PT domain structure of C1-C6 cyclization..**page 2**

Supplementary Fig. S3

The PT domain structure of C3-C8 cyclization..**page 3**

Supplementary Table S1

List of 661 NR-PKSs introduced in the phylogenic analysis...**page 4**

Supplementary Table S2

List of 55 NR-PKSs related to known polyketides...**page 21**

Supplementary Table S3

Structural prediction of PT domains...**page 27**

Supplementary Table S4

Information of PT domains...**page 29**

Supplementary Table S5

Conservation analysis of CLRs in PT domains...**page 30**

Figure S1. Phylogenetic analysis of 661 PT domains.

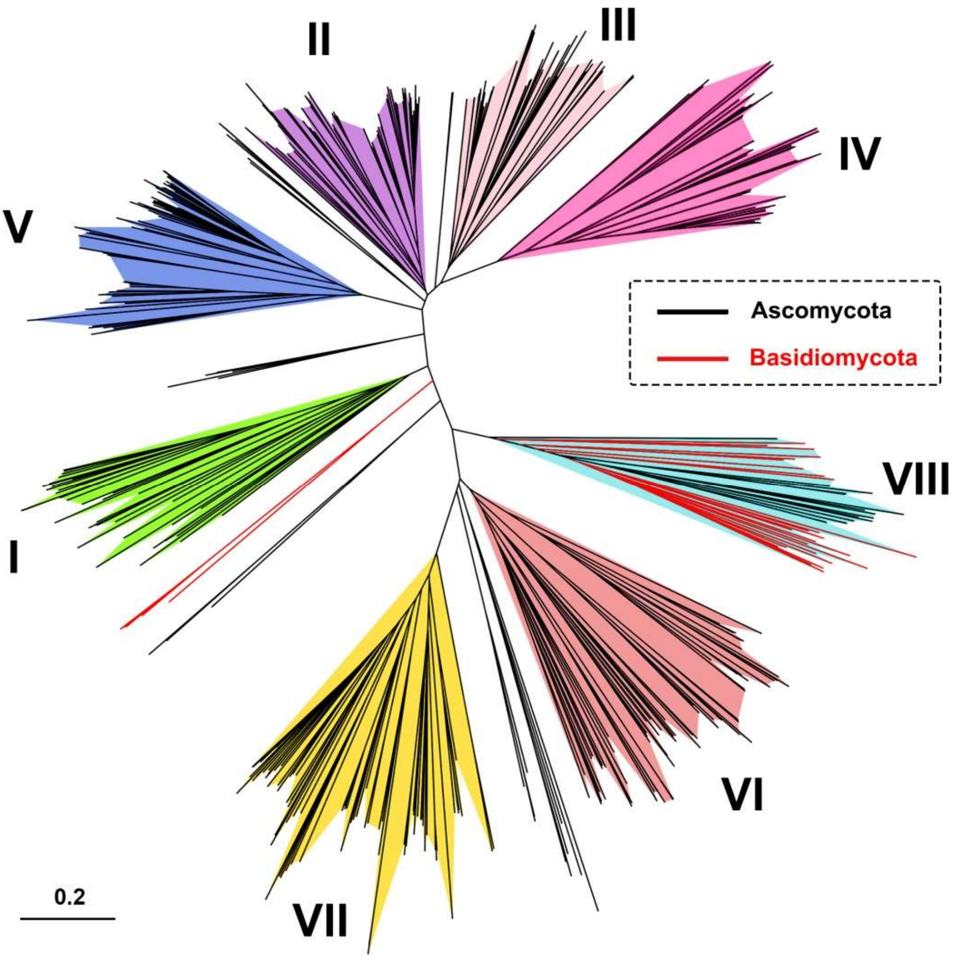

Figure S2. The PT domain structure of C1-C6 cyclization is exemplified by ADI24953 structural model in group V.

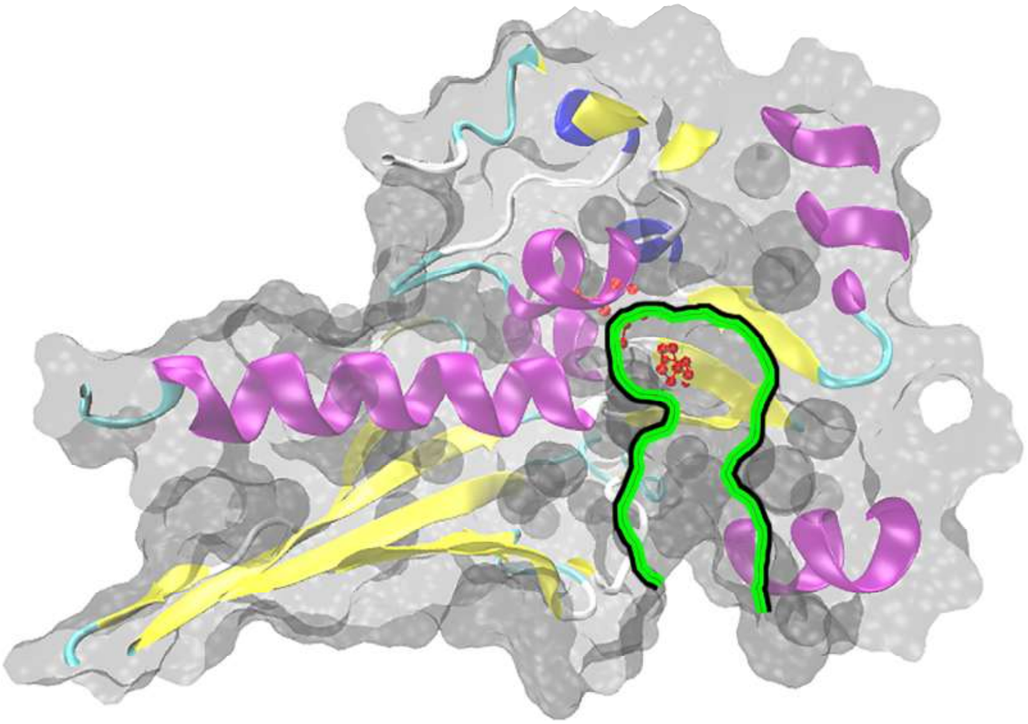

Figure S3. The PT domain structure of C3-C8 cyclization is exemplified by AGC95321 structural model in group I.

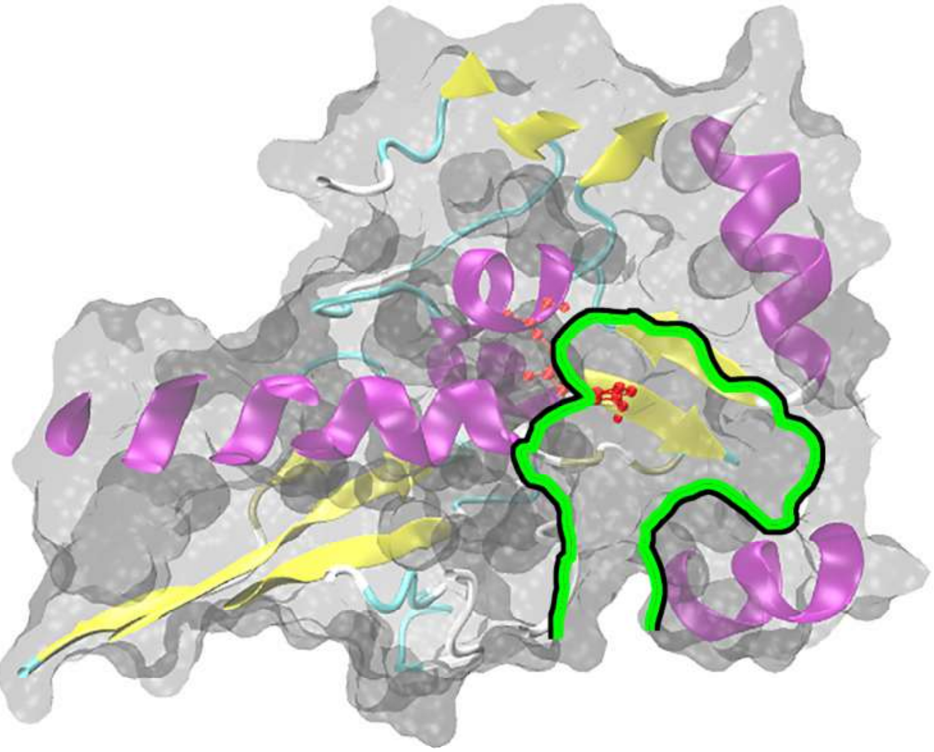

Table S1. List of 661 NR-PKSs introduced in the phylogenic analysis.

S/N	Group	Accession No.	Strain Name	Length (aa)
1	VIII	XP_006460038	*Agaricus bisporus var. bisporus* H97	1927
2	VIII	XP_007329045	*Agaricus bisporus var. burnettii* JB137-S8	1913
3	II	BAK64048	*Alternaria alternata*	2130
4	II	AEH76763	*Alternaria alternata*	2160
5	II	AFN68292	*Alternaria alternata*	2161
6	VIII	AFL91703	*Armillaria mellea*	2209
7	IV	XP_003013882	*Arthroderma benhamiae* CBS 112371	2140
8	V	XP_003013353	*Arthroderma benhamiae* CBS 112371	1798
9	VII	XP_003010590	*Arthroderma benhamiae* CBS 112371	2583
10	IV	XP_003169137	*Arthroderma gypseum* CBS 118893	2143
11	V	XP_003170602	*Arthroderma gypseum* CBS 118893	1797
12	VII	XP_003170502	*Arthroderma gypseum* CBS 118893	2583
13	III	XP_002847317	*Arthroderma otae* CBS 113480	2143
14	IV	XP_002842704	*Arthroderma otae* CBS 113480	2110
15	V	XP_002846705	*Arthroderma otae* CBS 113480	1794
16	V	XP_002848092	*Arthroderma otae* CBS 113480	1796
17	VI	XP_002843683	*Arthroderma otae* CBS 113480	2225
18	VII	XP_002846669	*Arthroderma otae* CBS 113480	2214
19	VII	XP_002848394	*Arthroderma otae* CBS 113480	2585
20	III	BAJ65431	*Aspergillus aculeatus*	2143
21	VI	XP_001273603	*Aspergillus clavatus* NRRL 1	1914
22	III	XP_001276035	*Aspergillus clavatus* NRRL 1	2145
23	V	XP_001275038	*Aspergillus clavatus* NRRL 1	1749
24	VII	XP_001273475	*Aspergillus clavatus* NRRL 1	2597
25	III	XP_002384329	*Aspergillus flavus* NRRL3357	2104
26	III	XP_002382817	*Aspergillus flavus* NRRL3357	2141
27	IV	XP_002373130	*Aspergillus flavus* NRRL3357	2045
28	IV	XP_002379951	*Aspergillus flavus* NRRL3357	2109
29	V	XP_002378746	*Aspergillus flavus* NRRL3357	1254
30	V	XP_002376725	*Aspergillus flavus* NRRL3357	1751
31	VII	XP_002379030	*Aspergillus flavus* NRRL3357	2245
32	VII	XP_002384603	*Aspergillus flavus* NRRL3357	2253
33	VII	XP_002384396	*Aspergillus flavus* NRRL3357	2580
34	VII	XP_002381902	*Aspergillus flavus* NRRL3357	2586
35	VIII	XP_002377153	*Aspergillus flavus* NRRL3357	2137
36	III	AAC39471	*Aspergillus fumigatus*	2146
37	III	EDP55264	*Aspergillus fumigatus* A1163	2146
38	V	EDP50840	*Aspergillus fumigatus* A1163	1741

39	V	EDP47078	*Aspergillus fumigatus* A1163	1776
40	V	EDP47964	*Aspergillus fumigatus* A1163	1794
41	VI	EDP48973	*Aspergillus fumigatus* A1163	1834
42	VIII	EDP49937	*Aspergillus fumigatus* A1163	2109
43	III	XP_756095	*Aspergillus fumigatus* Af293	2146
44	V	XP_746435	*Aspergillus fumigatus* Af293	1775
45	V	XP_751377	*Aspergillus fumigatus* Af293	1782
46	V	XP_746913	*Aspergillus fumigatus* Af293	1794
47	VI	XP_746971	*Aspergillus fumigatus* Af293	1834
48	VII	XP_748578	*Aspergillus fumigatus* Af293	2198
49	VI	GAA90162	*Aspergillus kawachii* IFO 4308	2585
50	III	GAA90949	*Aspergillus kawachii* IFO 4308	2123
51	III	GAA88246	*Aspergillus kawachii* IFO 4308	2136
52	V	GAA88581	*Aspergillus kawachii* IFO 4308	1745
53	V	GAA85937	*Aspergillus kawachii* IFO 4308	1797
54	VII	GAA92425	*Aspergillus kawachii* IFO 4308	2575
55	VII	GAA89817	*Aspergillus kawachii* IFO 4308	2621
56	III	BAK53402	*Aspergillus luchuensis*	2150
57	IV	AAA81586	*Aspergillus nidulans*	2181
58	VI	XP_664052	*Aspergillus nidulans* FGSC A4	2517
59	VII	XP_659636	*Aspergillus nidulans* FGSC A4	2221
60	VII	ANID_07903	*Aspergillus nidulans* FGSC A4	2605
61	I	XP_681178	*Aspergillus nidulans* FGSC A4	2103
62	III	Q03149	*Aspergillus nidulans* FGSC A4	2157
63	IV	Q12397	*Aspergillus nidulans* FGSC A4	2211
64	V	XP_663604	*Aspergillus nidulans* FGSC A4	1792
65	V	XP_657754	*Aspergillus nidulans* FGSC A4	1806
66	V	XP_664675	*Aspergillus nidulans* FGSC A4	1870
67	VI	XP_681652	*Aspergillus nidulans* FGSC A4	2476
68	VII	XP_660834	*Aspergillus nidulans* FGSC A4	2193
69	VII	XP_658127	*Aspergillus nidulans* FGSC A4	2476
70	VII	XP_660990	*Aspergillus nidulans* FGSC A4	2493
71	VII	XP_658638	*Aspergillus nidulans* FGSC A4	2793
72	III	EHA21301	*Aspergillus niger* ATCC 1015	2121
73	III	EHA28527	*Aspergillus niger* ATCC 1015	2128
74	V	EHA20150	*Aspergillus niger* ATCC 1015	1717
75	VI	EHA20911	*Aspergillus niger* ATCC 1015	2103
76	VII	EHA25844	*Aspergillus niger* ATCC 1015	2517
77	VII	EHA28237	*Aspergillus niger* ATCC 1015	2599
78	III	XP_001393884	*Aspergillus niger* CBS 513.88	2125
79	III	XP_001390425	*Aspergillus niger* CBS 513.88	2135

80	V	XP_001402309	*Aspergillus niger* CBS 513.88	1674
81	V	XP_001394705	*Aspergillus niger* CBS 513.88	1793
82	VI	XP_001393524	*Aspergillus niger* CBS 513.88	2620
83	VII	XP_001390084	*Aspergillus niger* CBS 513.88	2154
84	VII	XP_001395291	*Aspergillus niger* CBS 513.88	2518
85	VII	XP_001393501	*Aspergillus niger* CBS 513.88	2606
86	IV	AAS90047	*Aspergillus nomius*	2100
87	IV	ACH72912	*Aspergillus ochraceoroseus*	2199
88	IV	BAE71314	*Aspergillus oryzae*	2009
89	VII	KDE82080	*Aspergillus oryzae* 100-8	2580
90	III	EIT79828	*Aspergillus oryzae* 3.042	2141
91	IV	EIT81356	*Aspergillus oryzae* 3.042	2039
92	IV	EIT81872	*Aspergillus oryzae* 3.042	2049
93	V	EIT75060	*Aspergillus oryzae* 3.042	1722
94	V	EIT80443	*Aspergillus oryzae* 3.042	1890
95	VI	EIT79328	*Aspergillus oryzae* 3.042	2647
96	VII	EIT81451	*Aspergillus oryzae* 3.042	2245
97	VII	EIT75517	*Aspergillus oryzae* 3.042	2253
98	VII	EIT73076	*Aspergillus oryzae* 3.042	2456
99	VII	EIT82410	*Aspergillus oryzae* 3.042	2574
100	VIII	EIT78482	*Aspergillus oryzae* 3.042	2135
101	III	XP_001827098	*Aspergillus oryzae* RIB40	1938
102	III	XP_001822700	*Aspergillus oryzae* RIB40	2141
103	IV	XP_001817959	*Aspergillus oryzae* RIB40	2044
104	IV	XP_001821511	*Aspergillus oryzae* RIB40	2109
105	V	BAE58990	*Aspergillus oryzae* RIB40	1731
106	V	XP_001823362	*Aspergillus oryzae* RIB40	1891
107	VI	XP_001816639	*Aspergillus oryzae* RIB40	2654
108	VII	XP_001823618	*Aspergillus oryzae* RIB40	2245
109	VII	XP_001827405	*Aspergillus oryzae* RIB40	2253
110	VII	XP_001827158	*Aspergillus oryzae* RIB40	2361
111	VII	BAE56924	*Aspergillus oryzae* RIB40	2456
112	VII	XP_001818926	*Aspergillus oryzae* RIB40	2586
113	VIII	BAE59374	*Aspergillus oryzae* RIB40	2135
114	IV	Q12053	*Aspergillus parasiticus*	2109
115	–	EYE96821	*Aspergillus ruber* CBS 135680	2009
116	III	EYE97768	*Aspergillus ruber* CBS 135680	1858
117	III	EYE98507	*Aspergillus ruber* CBS 135680	2151
118	V	EYE98259	*Aspergillus ruber* CBS 135680	1767
119	VII	EYE91696	*Aspergillus ruber* CBS 135680	2595
120	IV	AAU08792	*Aspergillus sojae*	1847

121	IV	AAR32704	*Aspergillus* sp. L	2109
122	–	BAB88688	*Aspergillus terreus*	2157
123	I	AGC95321	*Aspergillus terreus*	2083
124	III	BAB88689	*Aspergillus terreus*	2187
125	V	BAB88752	*Aspergillus terreus*	1802
126	VI	XP_001209382	*Aspergillus terreus* NIH2624	2417
127	VII	XP_001212610	*Aspergillus terreus* NIH2624	2556
128	–	XP_001210231	*Aspergillus terreus* NIH2624	2081
129	III	XP_001216121	*Aspergillus terreus* NIH2624	1868
130	V	XP_001217072	*Aspergillus terreus* NIH2624	1771
131	V	XP_001211612	*Aspergillus terreus* NIH2624	1802
132	VI	XP_001212807	*Aspergillus terreus* NIH2624	2590
133	VII	XP_001217248	*Aspergillus terreus* NIH2624	2558
134	VII	XP_001216282	*Aspergillus terreus* NIH2624	2737
135	VIII	XP_001210065	*Aspergillus terreus* NIH2624	1777
136	IV	XP_007671613	*Baudoinia compniacensis* UAMH 10762	2242
137	III	EJP64619	*Beauveria bassiana* ARSEF 2860	2145
138	VIII	EJP62792	*Beauveria bassiana* ARSEF 2860	2211
139	V	ENH99769	*Bipolaris maydis* ATCC 48331	1393
140	II	EMD96875	*Bipolaris maydis* C5	2156
141	V	EMD89515	*Bipolaris maydis* C5	1314
142	V	EMD93898	*Bipolaris maydis* C5	1791
143	VI	EMD85328	*Bipolaris maydis* C5	2059
144	VI	EMD94543	*Bipolaris maydis* C5	2471
145	VII	EMD93081	*Bipolaris maydis* C5	2805
146	II	BAD22832	*Bipolaris oryzae*	2155
147	I	XP_007683625	*Bipolaris oryzae* ATCC 44560	1987
148	II	XP_007683730	*Bipolaris oryzae* ATCC 44560	2155
149	IV	XP_007688540	*Bipolaris oryzae* ATCC 44560	2266
150	V	XP_007688273	*Bipolaris oryzae* ATCC 44560	1784
151	V	XP_007692278	*Bipolaris oryzae* ATCC 44560	1802
152	VI	XP_007690544	*Bipolaris oryzae* ATCC 44560	2066
153	VI	XP_007689067	*Bipolaris oryzae* ATCC 44560	2716
154	VII	XP_007691023	*Bipolaris oryzae* ATCC 44560	2275
155	I	XP_007697886	*Bipolaris sorokiniana* ND90Pr	1990
156	II	XP_007696213	*Bipolaris sorokiniana* ND90Pr	2153
157	IV	XP_007696013	*Bipolaris sorokiniana* ND90Pr	2317
158	V	XP_007701226	*Bipolaris sorokiniana* ND90Pr	1791
159	V	XP_007696688	*Bipolaris sorokiniana* ND90Pr	1802
160	VII	XP_007700864	*Bipolaris sorokiniana* ND90Pr	2275
161	I	EUN21450	*Bipolaris victoriae* FI3	1993

162	II	EUN32222	*Bipolaris victoriae* FI3	2156
163	IV	EUN29146	*Bipolaris victoriae* FI3	2308
164	V	EUN25734	*Bipolaris victoriae* FI3	1779
165	VI	EUN22614	*Bipolaris victoriae* FI3	2064
166	VII	EUN22347	*Bipolaris victoriae* FI3	2583
167	I	XP_007712105	*Bipolaris zeicola* 26-R-13	1993
168	I	XP_007718130	*Bipolaris zeicola* 26-R-13	2091
169	II	XP_007706311	*Bipolaris zeicola* 26-R-13	2156
170	V	XP_007715766	*Bipolaris zeicola* 26-R-13	1788
171	VI	XP_007709531	*Bipolaris zeicola* 26-R-13	2043
172	VII	XP_007714759	*Bipolaris zeicola* 26-R-13	2583
173	V	CCU75801	*Blumeria graminis f. sp. hordei* DH14	1768
174	V	EPQ66189	*Blumeria graminis f. sp. tritici* 96224	1768
175	I	XP_001559596	*Botryotinia fuckeliana* B05.10	2055
176	II	XP_001554288	*Botryotinia fuckeliana* B05.10	2084
177	II	XP_001547095	*Botryotinia fuckeliana* B05.10	2138
178	V	XP_001553397	*Botryotinia fuckeliana* B05.10	1648
179	VI	XP_001550802	*Botryotinia fuckeliana* B05.10	2411
180	VII	XP_001559289	*Botryotinia fuckeliana* B05.10	2588
181	I	CCD53467	*Botryotinia fuckeliana* T4	2147
182	II	CCD52428	*Botryotinia fuckeliana* T4	2138
183	II	CCD50774	*Botryotinia fuckeliana* T4	2143
184	VI	CCD46020	*Botryotinia fuckeliana* T4	2129
185	VI	CCD50766	*Botryotinia fuckeliana* T4	2531
186	VI	CCD51172	*Botryotinia fuckeliana* T4	2676
187	VI	CCD56082	*Botryotinia fuckeliana* T4	2575
188	VII	CCD44950	*Botryotinia fuckeliana* T4	2585
189	I	EMR80535	*Botrytis cinerea* BcDW1	2147
190	II	EMR81302	*Botrytis cinerea* BcDW1	2138
191	II	EMR86678	*Botrytis cinerea* BcDW1	2159
192	V	EMR83380	*Botrytis cinerea* BcDW1	1755
193	VI	EMR89283	*Botrytis cinerea* BcDW1	2129
194	VI	EMR89251	*Botrytis cinerea* BcDW1	2676
195	VI	EMR86672	*Botrytis cinerea* BcDW1	2893
196	VI	EMR80977	*Botrytis cinerea* BcDW1	3089
197	–	GAD98443	*Byssochlamys spectabilis* No. 5	2112
198	II	GAD99561	*Byssochlamys spectabilis* No. 5	2037
199	VII	GAE00035	*Byssochlamys spectabilis* No. 5	2812
200	II	XP_007721308	*Capronia coronata* CBS 617.96	2163
201	VI	XP_007723599	*Capronia coronata* CBS 617.96	2542
202	II	XP_007728918	*Capronia epimyces* CBS 606.96	2164

203	V	XP_007731035	*Capronia epimyces* CBS 606.96	1855
204	VI	XP_007730098	*Capronia epimyces* CBS 606.96	2536
205	II	AAO60166	*Ceratocystis resinifera*	2188
206	IV	AAT69682	*Cercospora nicotianae*	2196
207	VIII	EMD42363	*Ceriporiopsis subvermispora* B	1979
208	I	ACM42403	*Chaetomium chiversii*	2138
209	II	XP_001219763	*Chaetomium globosum* CBS 148.51	2181
210	VI	XP_001226720	*Chaetomium globosum* CBS 148.51	2379
211	VI	XP_001228055	*Chaetomium globosum* CBS 148.51	2568
212	VII	XP_001227954	*Chaetomium globosum* CBS 148.51	2515
213	VII	XP_001227513	*Chaetomium globosum* CBS 148.51	2525
214	II	XP_006695455	*Chaetomium thermophilum var. thermophilum* DSM 1495	2231
215	–	ADM79459	*Cladonia grayi*	2089
216	–	ADX36086	*Cladonia grayi*	2155
217	–	ADX36085	*Cladonia grayi*	2161
218	II	ADX36087	*Cladonia grayi*	2146
219	VI	ADM79462	*Cladonia grayi*	2462
220	II	AFB81352	*Cladonia macilenta*	2146
221	–	ETI20206	*Cladophialophora carrionii* CBS 160.54	2150
222	II	ETI24557	*Cladophialophora carrionii* CBS 160.54	2176
223	V	ETI19899	*Cladophialophora carrionii* CBS 160.54	1788
224	II	XP_007740016	*Cladophialophora psammophila* CBS 110553	2180
225	III	XP_007740172	*Cladophialophora psammophila* CBS 110553	2230
226	II	XP_007756951	*Cladophialophora yegresii* CBS 114405	2177
227	V	XP_007754624	*Cladophialophora yegresii* CBS 114405	1791
228	V	scf7180000126987_G8647	*Cladosporium fulvum*	1790
229	II	AFP89389	*Cladosporium phlei*	2174
230	I	CCE33500	*Claviceps purpurea* 20.1	2228
231	V	CCE31584	*Claviceps purpurea* 20.1	1808
232	VI	CCE30403	*Claviceps purpurea* 20.1	2148
233	VI	XP_001243721	*Coccidioides immitis* RS	2126
234	VII	XP_001243185	*Coccidioides immitis* RS	2579
235	IV	XP_001241406	*Coccidioides immitis* RS	2267
236	VI	EAS33894	*Coccidioides immitis* RS	2250
237	VI	XP_001245248	*Coccidioides immitis* RS	2280
238	VII	XP_001246130	*Coccidioides immitis* RS	2220
239	IV	XP_003070496	*Coccidioides posadasii* C735 delta SOWgp	2242
240	VI	XP_003068966	*Coccidioides posadasii* C735 delta SOWgp	2203
241	VI	XP_003071593	*Coccidioides posadasii* C735 delta SOWgp	2250
242	VII	XP_003067742	*Coccidioides posadasii* C735 delta SOWgp	2225
243	VII	XP_003070229	*Coccidioides posadasii* C735 delta SOWgp	2614

244	IV	EFW18011	*Coccidioides posadasii* str. Silveira	2258
245	VI	EFW13280	*Coccidioides posadasii* str. Silveira	2070
246	VI	EFW20973	*Coccidioides posadasii* str. Silveira	2211
247	VII	EFW17356	*Coccidioides posadasii* str. Silveira	2205
248	VII	EFW23245	*Coccidioides posadasii* str. Silveira	2611
249	–	XP_007598227	*Colletotrichum fioriniae* PJ7	1892
250	I	XP_007589869	*Colletotrichum fioriniae* PJ7	2106
251	II	XP_007596282	*Colletotrichum fioriniae* PJ7	2181
252	III	XP_007599710	*Colletotrichum fioriniae* PJ7	2120
253	IV	XP_007592254	*Colletotrichum fioriniae* PJ7	2115
254	VI	XP_007596699	*Colletotrichum fioriniae* PJ7	2332
255	–	EQB53326	*Colletotrichum gloeosporioides* Cg-14	1931
256	I	EQB52988	*Colletotrichum gloeosporioides* Cg-14	2191
257	II	EQB55056	*Colletotrichum gloeosporioides* Cg-14	2178
258	IV	EQB52152	*Colletotrichum gloeosporioides* Cg-14	2114
259	VI	EQB55260	*Colletotrichum gloeosporioides* Cg-14	1241
260	VI	EQB51125	*Colletotrichum gloeosporioides* Cg-14	2028
261	VIII	EQB49011	*Colletotrichum gloeosporioides* Cg-14	2031
262	VI	XP_007284106	*Colletotrichum gloeosporioides* Nara gc5	2110
263	I	XP_007280259	*Colletotrichum gloeosporioides* Nara gc5	2089
264	II	XP_007287024	*Colletotrichum gloeosporioides* Nara gc5	2156
265	IV	XP_007281131	*Colletotrichum gloeosporioides* Nara gc5	2115
266	VI	XP_007283523	*Colletotrichum gloeosporioides* Nara gc5	2066
267	–	EFQ28363	*Colletotrichum graminicola* M1.001	2157
268	I	EFQ36690	*Colletotrichum graminicola* M1.001	2086
269	I	EFQ36633	*Colletotrichum graminicola* M1.001	2241
270	II	EFQ29059	*Colletotrichum graminicola* M1.001	2178
271	IV	EFQ33691	*Colletotrichum graminicola* M1.001	2116
272	IV	EFQ36810	*Colletotrichum graminicola* M1.001	2295
273	V	EFQ33703	*Colletotrichum graminicola* M1.001	1777
274	VI	EFQ36407	*Colletotrichum graminicola* M1.001	2510
275	VI	EFQ36652	*Colletotrichum graminicola* M1.001	2669
276	VII	EFQ36707	*Colletotrichum graminicola* M1.001	2355
277	VII	EFQ34124	*Colletotrichum graminicola* M1.001	2633
278	I	CCF46696	*Colletotrichum higginsianum*	1871
279	II	CCF45141	*Colletotrichum higginsianum*	2180
280	IV	CCF41085	*Colletotrichum higginsianum*	1706
281	VI	CCF43671	*Colletotrichum higginsianum*	2449
282	VI	CCF32372	*Colletotrichum higginsianum*	1820
283	VII	CCF39467	*Colletotrichum higginsianum*	1626
284	II	BAA18956	*Colletotrichum lagenaria*	2187

285	–	ENH82422	*Colletotrichum orbiculare* MAFF 240422	2490
286	I	ENH86452	*Colletotrichum orbiculare* MAFF 240422	2245
287	I	ENH83455	*Colletotrichum orbiculare* MAFF 240422	2283
288	II	ENH81867	*Colletotrichum orbiculare* MAFF 240422	2183
289	IV	ENH84744	*Colletotrichum orbiculare* MAFF 240422	2145
290	IV	ENH81662	*Colletotrichum orbiculare* MAFF 240422	2152
291	VI	ENH84250	*Colletotrichum orbiculare* MAFF 240422	2538
292	VIII	ENH82425	*Colletotrichum orbiculare* MAFF 240422	1984
293	VIII	XP_007775190	*Coniophora_puteana*_RWD-64-598_SS2	2216
294	–	EON61283	*Coniosporium apollinis* CBS 100218	2118
295	II	EON65175	*Coniosporium apollinis* CBS 100218	2172
296	III	EON65343	*Coniosporium apollinis* CBS 100218	2141
297	VIII	XP_001835415	*Coprinopsis cinerea* okayama7#130	1728
298	III	XP_006668876	*Cordyceps militaris* CM01	2158
299	IV	XP_006667138	*Cordyceps militaris* CM01	2218
300	II	ETN44617	*Cyphellophora europaea* CBS 101466	2171
301	II	ADP05113	*Daldinia eschscholtzii*	2175
302	VIII	XP_007368302	*Dichomitus squalens* LYAD-421 SS1	1718
303	II	ACH72076	*Dirinaria applanata*	1882
304	II	ABS85549	*Dothiorella aegiceri*	1780
305	II	EME39782	*Dothistroma septosporum* NZE10	2189
306	II	ABU63483	*Elsinoe fawcettii*	2192
307	II	XP_007804330	*Endocarpon pusillum* Z07020	2145
308	III	XP_007785431	*Endocarpon pusillum* Z07020	2164
309	V	XP_007786761	*Endocarpon pusillum* Z07020	1583
310	VII	XP_007800756	*Endocarpon pusillum* Z07020	2306
311	VII	XP_007801017	*Endocarpon pusillum* Z07020	2691
312	II	XP_007796413	*Eutypa lata* UCREL1	1701
313	V	XP_007793674	*Eutypa lata* UCREL1	1718
314	VI	XP_007790426	*Eutypa lata* UCREL1	2508
315	VII	XP_007793245	*Eutypa lata* UCREL1	2386
316	VII	XP_007793864	*Eutypa lata* UCREL1	2408
317	II	AAD31436	*Exophiala dermatitidis*	2177
318	–	AAN74983	*Exophiala lecanii-corni*	2155
319	II	AAN75188	*Exophiala lecanii-corni*	2192
320	VIII	CCM00101	*Fibroporia radiculosa*	1996
321	VIII	XP_007262597	*Fomitiporia mediterranea* MF3/22	2269
322	VIII	EPS94471	*Fomitopsis pinicola* FP-58527 SS1	2091
323	III	CAB92399	*Fusarium fujikuroi*	2009
324	IV	CAC88775	*Fusarium fujikuroi*	1317
325	IV	CCE67070	*Fusarium fujikuroi*	2286

326	VII	CCT76054	*Fusarium fujikuroi* IMI 58289	2248
327	I	ABB90282	*Fusarium graminearum*	2036
328	III	AAU10633	*Fusarium graminearum*	2073
329	IV	EYB26831	*Fusarium graminearum*	2287
330	VIII	EYB32182	*Fusarium graminearum*	2029
331	III	ESU07748	*Fusarium graminearum* PH-1	2067
332	IV	ESU15719	*Fusarium graminearum* PH-1	2138
333	VI	XP_384764	*Fusarium graminearum* PH-1	2172
334	VIII	XP_384140	*Fusarium graminearum* PH-1	2029
335	IV	EXL68993	*Fusarium oxysporum f. sp. conglutinans race* 2 54008	2286
336	III	ENH68137	*Fusarium oxysporum f. sp. cubense race* 1	1731
337	IV	ENH69483	*Fusarium oxysporum f. sp. cubense race* 1	2163
338	III	EMT69321	*Fusarium oxysporum f. sp. cubense race* 4	2111
339	III	EXL92030	*Fusarium oxysporum f. sp. cubense tropical race* 4 54006	2036
340	IV	EXM03524	*Fusarium oxysporum f. sp. cubense tropical race* 4 54006	2285
341	III	EWZ80090	*Fusarium oxysporum f. sp. lycopersici* MN25	2036
342	IV	EWZ99873	*Fusarium oxysporum f. sp. lycopersici* MN25	2285
343	III	EXK38465	*Fusarium oxysporum f. sp. melonis* 26406	2036
344	IV	EXK32837	*Fusarium oxysporum f. sp. melonis* 26406	2285
345	III	EXA44005	*Fusarium oxysporum f. sp. pisi* HDV247	2036
346	IV	EXA46686	*Fusarium oxysporum f. sp. pisi* HDV247	2285
347	III	EXL55894	*Fusarium oxysporum f. sp. radicis-lycopersici* 26381	2036
348	IV	EXL58547	*Fusarium oxysporum f. sp. radicis-lycopersici* 26381	2285
349	III	EXK83376	*Fusarium oxysporum f. sp. raphani* 54005	2036
350	IV	EXK80558	*Fusarium oxysporum f. sp. raphani* 54005	2285
351	III	EXM24735	*Fusarium oxysporum f. sp. vasinfectum* 25433	2036
352	IV	EXM20110	*Fusarium oxysporum f. sp. vasinfectum* 25433	2285
353	III	EWZ36433	*Fusarium oxysporum* Fo47	2034
354	IV	EWZ43044	*Fusarium oxysporum* Fo47	2285
355	IV	EGU74198	*Fusarium oxysporum* Fo5176	2284
356	III	EWY89850	*Fusarium oxysporum* FOSC 3-a	2036
357	IV	EWY95164	*Fusarium oxysporum* FOSC 3-a	2287
358	I	EKJ72968	*Fusarium pseudograminearum* CS3096	2036
359	III	EKJ76027	*Fusarium pseudograminearum* CS3096	2072
360	IV	EKJ76363	*Fusarium pseudograminearum* CS3096	2287
361	VI	EKJ73816	*Fusarium pseudograminearum* CS3096	2169
362	VIII	EKJ74560	*Fusarium pseudograminearum* CS3096	2373

363	III	EWG41233	*Fusarium verticillioides* 7600	2036
364	IV	EWG41617	*Fusarium verticillioides* 7600	2272
365	II	EJT80408	*Gaeumannomyces graminis var. tritici* R3-111a-1	2168
366	V	EJT69423	*Gaeumannomyces graminis var. tritici* R3-111a-1	1953
367	II	AAN59953	*Glarea lozoyensis*	2124
368	VI	EHK97154	*Glarea lozoyensis* 74030	1906
369	VII	EHK98549	*Glarea lozoyensis* 74030	2009
370	I	EHK97626	*Glarea lozoyensis* 74030	2052
371	II	EHL00222	*Glarea lozoyensis* 74030	2107
372	I	EPE35573	*Glarea lozoyensis* ATCC 20868	2077
373	I	EPE32109	*Glarea lozoyensis* ATCC 20868	2170
374	II	EPE33704	*Glarea lozoyensis* ATCC 20868	1988
375	II	EPE34890	*Glarea lozoyensis* ATCC 20868	2129
376	IV	EPE29990	*Glarea lozoyensis* ATCC 20868	2235
377	VI	EPE27454	*Glarea lozoyensis* ATCC 20868	2482
378	VI	EPE28832	*Glarea lozoyensis* ATCC 20868	2497
379	VII	EPE28681	*Glarea lozoyensis* ATCC 20868	2700
380	VIII	XP_007866703	*Gloeophyllum_trabeum*_ATCC_11539	2006
381	VIII	BAO20284	*Grifola frondosa*	2103
382	VII	EFX04833	*Grosmannia clavigera* kw1407	2664
383	II	EFX02617	*Grosmannia clavigera* kw1407	2173
384	IV	EFX00827	*Grosmannia clavigera* kw1407	2099
385	V	EFX04268	*Grosmannia clavigera* kw1407	1697
386	VIII	ETW82343	*Heterobasidion irregulare* TC 32-1	2025
387	VIII	ETW82342	*Heterobasidion irregulare* TC 32-1	2237
388	VII	AFO67256	*Hypogymnia physodes*	2394
389	VII	AFO67255	*Hypogymnia physodes*	2369
390	I	ACD39762	*Hypomyces subiculosus*	2049
391	VIII	XP_001876029	*Laccaria bicolor* S238N-H82	1994
392	I	AHV78247	*Lasiodiplodia theobromae*	2083
393	IV	AAS92537	*Leptosphaeria maculans*	2028
394	II	XP_003841919	*Leptosphaeria maculans* JN3	2165
395	IV	XP_003842432	*Leptosphaeria maculans* JN3	2046
396	VII	XP_003842913	*Leptosphaeria maculans* JN3	1899
397	VII	EKG11417	*Macrophomina phaseolina* MS6	1329
398	V	EKG18431	*Macrophomina phaseolina* MS6	1767
399	VI	EKG13736	*Macrophomina phaseolina* MS6	2214
400	VI	EKG19938	*Macrophomina phaseolina* MS6	2257
401	VI	EKG10413	*Macrophomina phaseolina* MS6	2607
402	VI	EKG13586	*Macrophomina phaseolina* MS6	2275

403	VI	EKG18751	*Macrophomina phaseolina* MS6	2550
404	VIII	EKG09551	*Macrophomina phaseolina* MS6	2110
405	I	XP_003715799	*Magnaporthe oryzae* 70-15	1860
406	I	XP_003718865	*Magnaporthe oryzae* 70-15	2152
407	II	XP_003715434	*Magnaporthe oryzae* 70-15	2181
408	IV	XP_003718644	*Magnaporthe oryzae* 70-15	2173
409	VII	XP_003717493	*Magnaporthe oryzae* 70-15	2307
410	VII	ELQ61531	*Magnaporthe oryzae* P131	2237
411	IV	ELQ68526	*Magnaporthe oryzae* P131	2121
412	I	ELQ42257	*Magnaporthe oryzae* Y34	2069
413	II	ELQ39536	*Magnaporthe oryzae* Y34	2149
414	IV	ELQ42604	*Magnaporthe oryzae* Y34	2173
415	VII	ELQ38934	*Magnaporthe oryzae* Y34	2251
416	I	XP_007294614	*Marssonina brunnea f. sp. 'multigermtubi'* MB_m1	2089
417	II	XP_007295542	*Marssonina brunnea f. sp. 'multigermtubi'* MB_m1	2162
418	II	XP_007288149	*Marssonina brunnea f. sp. 'multigermtubi'* MB_m1	2926
419	I	ACD39770	*Metacordyceps chlamydosporia*	2090
420	III	XP_007815650	*Metarhizium acridum* CQMa 102	1861
421	III	XP_007807862	*Metarhizium acridum* CQMa 102	1925
422	III	XP_007811725	*Metarhizium acridum* CQMa 102	2165
423	III	XP_007806517	*Metarhizium acridum* CQMa 102	2200
424	V	XP_007810456	*Metarhizium acridum* CQMa 102	1798
425	VI	XP_007814547	*Metarhizium acridum* CQMa 102	2183
426	VII	XP_007816051	*Metarhizium acridum* CQMa 102	2571
427	VII	XP_007810891	*Metarhizium acridum* CQMa 102	2627
428	I	XP_007824738	*Metarhizium anisopliae* ARSEF 23	1767
429	III	XP_007819428	*Metarhizium anisopliae* ARSEF 23	2046
430	III	XP_007823934	*Metarhizium anisopliae* ARSEF 23	2083
431	III	XP_007826081	*Metarhizium anisopliae* ARSEF 23	2186
432	III	XP_007824559	*Metarhizium anisopliae* ARSEF 23	2216
433	V	XP_007825109	*Metarhizium anisopliae* ARSEF 23	1789
434	V	XP_007822764	*Metarhizium anisopliae* ARSEF 23	1795
435	VI	XP_007823685	*Metarhizium anisopliae* ARSEF 23	2181
436	VII	XP_007826136	*Metarhizium anisopliae* ARSEF 23	2335
437	VII	XP_007824114	*Metarhizium anisopliae* ARSEF 23	2627
438	VII	EXU95978	*Metarhizium robertsii*	2333
439	I	EXU99173	*Metarhizium robertsii*	1706
440	III	EXU98524	*Metarhizium robertsii*	2088
441	III	EXU95796	*Metarhizium robertsii*	2148

442	III	EXV02536	*Metarhizium robertsii*	2151
443	VII	AGN71604	*Monascus pilosus*	2689
444	II	CAC94008	*Monascus purpureus*	2061
445	VII	BAD44749	*Monascus purpureus*	2584
446	VIII	XP_007844893	*Moniliophthora_roreri*_MCA_2997	2020
447	VIII	XP_007850276	*Moniliophthora_roreri*_MCA_2997	2132
448	VIII	XP_007850268	*Moniliophthora_roreri*_MCA_2997	2152
449	VIII	XP_007844087	*Moniliophthora_roreri*_MCA_2997	2201
450	II	XP_003661126	*Myceliophthora thermophila* ATCC 42464	2234
451	III	XP_003666434	*Myceliophthora thermophila* ATCC 42464	2356
452	V	XP_003663601	*Myceliophthora thermophila* ATCC 42464	1807
453	VIII	XP_003664878	*Myceliophthora thermophila* ATCC 42464	2268
454	IV	ADO14690	*Mycosphaerella coffeicola*	2195
455	IV	AAZ95017	*Mycosphaerella pini*	2399
456	III	AAS48892	*Nectria haematococca*	2106
457	III	XP_003045916	*Nectria haematococca* mpVI 77-13-4	2106
458	IV	XP_003039929	*Nectria haematococca* mpVI 77-13-4	2298
459	–	XP_007587223	*Neofusicoccum parvum* UCRNP2	1746
460	II	XP_007584358	*Neofusicoccum parvum* UCRNP2	2169
461	VII	XP_007579490	*Neofusicoccum parvum* UCRNP2	2538
462	III	XP_001261235	*Neosartorya fischeri* NRRL 181	2144
463	V	XP_001267621	*Neosartorya fischeri* NRRL 181	1777
464	V	XP_001266594	*Neosartorya fischeri* NRRL 181	1785
465	V	XP_001262597	*Neosartorya fischeri* NRRL 181	1794
466	V	XP_001266579	*Neosartorya fischeri* NRRL 181	1821
467	VI	XP_001261880	*Neosartorya fischeri* NRRL 181	1816
468	VII	XP_001258915	*Neosartorya fischeri* NRRL 181	2620
469	II	XP_960586	*Neurospora crassa* OR74A	2083
470	II	EAA31350	*Neurospora crassa* OR74A	2206
471	II	EGO61342	*Neurospora tetrasperma* FGSC 2508	2205
472	II	EGZ74641	*Neurospora tetrasperma* FGSC 2509	2194
473	II	AAD38786	*Nodulisporium sp.* ATCC74245	2159
474	II	ABD47522	*Ophiostoma piceae*	2180
475	–	EPE10458	*Ophiostoma piceae* UAMH 11346	2248
476	IV	ADN06232	*Passalora arachidicola*	1959
477	II	AEE65374	*Peltigera membranacea*	2125
478	II	AEE65372	*Peltigera membranacea*	2138
479	V	ADI24953	*Penicillium aethiopicum*	1790
480	V	ADI24926	*Penicillium aethiopicum*	1824
481	VI	ADY00130	*Penicillium brevicompactum*	2448
482	VII	XP_002567553	*Penicillium chrysogenum* Wisconsin 54-1255	2664

483	I	XP_002568275	*Penicillium chrysogenum* Wisconsin 54-1255	2098
484	III	XP_002568608	*Penicillium chrysogenum* Wisconsin 54-1255	2138
485	III	EKV10885	*Penicillium digitatum* PHI26	2138
486	VI	EKV16250	*Penicillium digitatum* PHI26	2294
487	VII	EKV06857	*Penicillium digitatum* PHI26	2554
488	III	EPS34527	*Penicillium oxalicum* 114-2	2141
489	V	EPS34273	*Penicillium oxalicum* 114-2	1786
490	III	CDM29654	*Penicillium roqueforti*	2136
491	VI	CDM36726	*Penicillium roqueforti*	2477
492	VI	CDM27382	*Penicillium roqueforti*	2495
493	II	XP_007833873	*Pestalotiopsis fici* W106-1	2155
494	IV	XP_007833333	*Pestalotiopsis fici* W106-1	2313
495	V	XP_007837596	*Pestalotiopsis fici* W106-1	1784
496	VI	XP_007830758	*Pestalotiopsis fici* W106-1	1933
497	VI	XP_007827138	*Pestalotiopsis fici* W106-1	2348
498	VI	XP_007836860	*Pestalotiopsis fici* W106-1	2512
499	II	AGT56219	*Pestalotiopsis malicola*	2155
500	II	XP_001802212	*Phaeosphaeria nodorum* SN15	2144
501	III	XP_001798591	*Phaeosphaeria nodorum* SN15	2234
502	IV	XP_001798923	*Phaeosphaeria nodorum* SN15	1906
503	V	XP_001805964	*Phaeosphaeria nodorum* SN15	1505
504	VI	XP_001797044	*Phaeosphaeria nodorum* SN15	2262
505	VI	XP_001797375	*Phaeosphaeria nodorum* SN15	2460
506	VIII	XP_007398509	*Phanerochaete carnosa* HHB-10118-sp	1806
507	VI	XP_001906796	*Podospora anserina* S mat+	2441
508	–	XP_001911464	*Podospora anserina* S mat+	2119
509	II	XP_001910795	*Podospora anserina* S mat+	2167
510	IV	XP_001911528	*Podospora anserina* S mat+	2263
511	VI	XP_001905653	*Podospora anserina* S mat+	2276
512	VIII	XP_001903585	*Podospora anserina* S mat+	1858
513	II	XP_007931490	*Pseudocercospora fijiensis* CIRAD86	2212
514	V	XP_007929626	*Pseudocercospora fijiensis* CIRAD86	1762
515	II	ELR06213	*Pseudogymnoascus destructans* 20631-21	2203
516	III	ELR10234	*Pseudogymnoascus destructans* 20631-21	2073
517	III	ELR07706	*Pseudogymnoascus destructans* 20631-21	2132
518	V	ELR08155	*Pseudogymnoascus destructans* 20631-21	1805
519	VII	ELR07152	*Pseudogymnoascus destructans* 20631-21	2670
520	–	ETS61551	*Pseudozyma aphidis* DSM 70725	1969
521	–	EST06961	*Pseudozyma brasiliensis*	1973
522	–	GAC94989	*Pseudozyma hubeiensis* SY62	1977
523	–	XP_007879461	*Pseudozyma_flocculosa*_PF-1	1947

524	VIII	XP_007386370	*Punctularia strigosozonata* HHB-11173 SS5	2108
525	II	XP_003298917	*Pyrenophora teres f. teres* 0-1	2150
526	IV	XP_003306836	*Pyrenophora teres f. teres* 0-1	2306
527	V	XP_003296346	*Pyrenophora teres f. teres* 0-1	1785
528	VI	XP_003301870	*Pyrenophora teres f. teres* 0-1	2548
529	VI	XP_003300445	*Pyrenophora teres f. teres* 0-1	2699
530	VII	XP_003299580	*Pyrenophora teres f. teres* 0-1	2589
531	I	XP_001934718	*Pyrenophora tritici-repentis* Pt-1C-BFP	1981
532	II	XP_001933656	*Pyrenophora tritici-repentis* Pt-1C-BFP	2106
533	V	XP_001933041	*Pyrenophora tritici-repentis* Pt-1C-BFP	1788
534	VI	XP_001937752	*Pyrenophora tritici-repentis* Pt-1C-BFP	2372
535	VIII	XP_001939987	*Pyrenophora tritici-repentis* Pt-1C-BFP	2036
536	VIII	CCX04910	*Pyronema omphalodes* CBS 100304	2087
537	II	BAO58974	*Rosellinia necatrix*	2160
538	II	ABG91136	*Rusavskia elegans*	2144
539	VII	CAN87161	*Sarocladium strictum*	2729
540	I	AHV78253	*Sarocladium zeae*	2211
541	VIII	XP_003038401	*Schizophyllum commune* H4-8	1917
542	II	ESZ91707	*Sclerotinia borealis* F-4157	2138
543	II	ESZ92457	*Sclerotinia borealis* F-4157	2143
544	IV	ESZ99281	*Sclerotinia borealis* F-4157	2245
545	V	ESZ98980	*Sclerotinia borealis* F-4157	1771
546	VI	ESZ91464	*Sclerotinia borealis* F-4157	2571
547	II	XP_001585805	*Sclerotinia sclerotiorum* 1980 UF-70	2138
548	II	XP_001586760	*Sclerotinia sclerotiorum* 1980 UF-70	2144
549	IV	XP_001592760	*Sclerotinia sclerotiorum* 1980 UF-70	1820
550	VIII	EGN93845	*Serpula lacrymans var. lacrymans* S7.3	2052
551	VIII	XP_007323934	*Serpula lacrymans var. lacrymans* S7.9	2110
552	–	EOA85543	*Setosphaeria turcica* Et28A	2126
553	I	EOA91583	*Setosphaeria turcica* Et28A	2033
554	I	EOA86107	*Setosphaeria turcica* Et28A	2261
555	II	EOA89647	*Setosphaeria turcica* Et28A	2158
556	IV	EOA85517	*Setosphaeria turcica* Et28A	2330
557	V	EOA88807	*Setosphaeria turcica* Et28A	1796
558	VI	EOA83736	*Setosphaeria turcica* Et28A	2541
559	VI	EOA85421	*Setosphaeria turcica* Et28A	2702
560	II	CAM35471	*Sordaria macrospora*	2205
561	II	EMF09017	*Sphaerulina musiva* SO2202	2190
562	V	EMF17386	*Sphaerulina musiva* SO2202	1787
563	VI	EMF17516	*Sphaerulina musiva* SO2202	2240
564	VIII	EMF08276	*Sphaerulina musiva* SO2202	2047

565	–	CBQ71604	*Sporisorium reilianum* SRZ2	1974
566	–	ERS97304	*Sporothrix schenckii* ATCC 58251	2228
567	II	ERS95912	*Sporothrix schenckii* ATCC 58251	2212
568	VIII	EIM84127	*Stereum hirsutum* FP-91666 SS1	2220
569	VIII	XP_007300189	*Stereum hirsutum* FP-91666 SS1	2407
570	VII	ADH01671	*Talaromyces marneffei*	2575
571	–	XP_002152334	*Talaromyces marneffei* ATCC 18224	2134
572	I	XP_002151003	*Talaromyces marneffei* ATCC 18224	2039
573	I	XP_002146110	*Talaromyces marneffei* ATCC 18224	2060
574	II	XP_002149119	*Talaromyces marneffei* ATCC 18224	2142
575	III	XP_002147717	*Talaromyces marneffei* ATCC 18224	2138
576	III	XP_002145792	*Talaromyces marneffei* ATCC 18224	2171
577	V	XP_002149615	*Talaromyces marneffei* ATCC 18224	1754
578	V	XP_002144865	*Talaromyces marneffei* ATCC 18224	1808
579	VI	XP_002145720	*Talaromyces marneffei* ATCC 18224	2225
580	VI	XP_002149742	*Talaromyces marneffei* ATCC 18224	1806
581	VII	XP_002149769	*Talaromyces marneffei* ATCC 18224	2683
582	VI	XP_002487764	*Talaromyces stipitatus* ATCC 10500	2012
583	–	XP_002483594	*Talaromyces stipitatus* ATCC 10500	2125
584	I	XP_002488697	*Talaromyces stipitatus* ATCC 10500	2200
585	III	XP_002481882	*Talaromyces stipitatus* ATCC 10500	2142
586	III	XP_002478062	*Talaromyces stipitatus* ATCC 10500	2157
587	IV	XP_002483004	*Talaromyces stipitatus* ATCC 10500	2051
588	V	XP_002482902	*Talaromyces stipitatus* ATCC 10500	1786
589	V	XP_002482968	*Talaromyces stipitatus* ATCC 10500	1809
590	VI	XP_002339967	*Talaromyces stipitatus* ATCC 10500	2635
591	VI	XP_002340065	*Talaromyces stipitatus* ATCC 10500	1879
592	VII	XP_002340070	*Talaromyces stipitatus* ATCC 10500	2574
593	VII	XP_002487778	*Talaromyces stipitatus* ATCC 10500	2606
594	VII	XP_002481993	*Talaromyces stipitatus* ATCC 10500	2661
595	VII	XP_002340038	*Talaromyces stipitatus* ATCC 10500	2664
596	VIII	XP_002478017	*Talaromyces stipitatus* ATCC 10500	1989
597	VIII	XP_002486858	*Talaromyces stipitatus* ATCC 10500	2104
598	II	XP_003648614	*Thielavia terrestris* NRRL 8126	2183
599	IV	XP_003656937	*Thielavia terrestris* NRRL 8126	2057
600	V	XP_003652581	*Thielavia terrestris* NRRL 8126	1807
601	VI	XP_003653746	*Thielavia terrestris* NRRL 8126	2092
602	VII	XP_003651141	*Thielavia terrestris* NRRL 8126	2483
603	VII	XP_003653735	*Thielavia terrestris* NRRL 8126	2638
604	VII	XP_003654933	*Thielavia terrestris* NRRL 8126	2670
605	II	XP_007912544	*Togninia minima* UCRPA7	2172

606	VII	XP_007915823	*Togninia minima* UCRPA7	2729
607	VIII	EIW56045	*Trametes versicolor* FP-101664 SS1	1768
608	I	EHK49847	*Trichoderma atroviride* IMI 206040	2084
609	III	EHK46843	*Trichoderma atroviride* IMI 206040	2147
610	V	EHK46042	*Trichoderma atroviride* IMI 206040	1818
611	VI	EHK42299	*Trichoderma atroviride* IMI 206040	2113
612	VII	EHK44445	*Trichoderma atroviride* IMI 206040	2697
613	I	AEX63665	*Trichoderma harzianum*	1720
614	III	XP_006969537	*Trichoderma reesei* QM6a	2146
615	V	XP_006969240	*Trichoderma reesei* QM6a	1863
616	VI	XP_006964226	*Trichoderma reesei* QM6a	2116
617	VII	XP_006961156	*Trichoderma reesei* QM6a	2633
618	VI	ETS03185	*Trichoderma reesei* RUT C-30	2110
619	I	EHK16308	*Trichoderma virens* Gv29-8	1736
620	III	EHK20748	*Trichoderma virens* Gv29-8	2147
621	V	EHK20655	*Trichoderma virens* Gv29-8	1884
622	VI	EHK17233	*Trichoderma virens* Gv29-8	1742
623	VI	EHK23001	*Trichoderma virens* Gv29-8	2146
624	III	EGE06072	*Trichophyton equinum* CBS 127.97	1440
625	IV	EGE06793	*Trichophyton equinum* CBS 127.97	2041
626	V	EGE06343	*Trichophyton equinum* CBS 127.97	1777
627	VII	EGE04288	*Trichophyton equinum* CBS 127.97	2584
628	IV	EZF36469	*Trichophyton interdigitale* H6	2128
629	VII	EZF36181	*Trichophyton interdigitale* H6	2583
630	IV	KDB24468	*Trichophyton interdigitale* MR816	2128
631	V	KDB28089	*Trichophyton interdigitale* MR816	1802
632	IV	XP_003235798	*Trichophyton rubrum* CBS 118892	2140
633	V	XP_003231059	*Trichophyton rubrum* CBS 118892	1798
634	VII	XP_003234762	*Trichophyton rubrum* CBS 118892	2584
635	V	EZG11077	*Trichophyton rubrum* CBS 735.88	1798
636	VII	EZG06712	*Trichophyton rubrum* CBS 735.88	2209
637	VII	EZF23313	*Trichophyton rubrum* MR850	2208
638	V	EZF78756	*Trichophyton soudanense* CBS 452.61	1798
639	VII	EZF74361	*Trichophyton soudanense* CBS 452.61	2208
640	III	EGD96701	*Trichophyton tonsurans* CBS 112818	2106
641	IV	EGD94040	*Trichophyton tonsurans* CBS 112818	2140
642	V	EGD99348	*Trichophyton tonsurans* CBS 112818	1802
643	VII	EGD97507	*Trichophyton tonsurans* CBS 112818	2584
644	IV	XP_003021298	*Trichophyton verrucosum* HKI 0517	2040
645	V	XP_003025446	*Trichophyton verrucosum* HKI 0517	1819
646	VII	XP_003023417	*Trichophyton verrucosum* HKI 0517	2584

647	VI	XP_002542778	*Uncinocarpus reesii* 1704	2149
648	I	AGI60158	*Usnea longissima*	2072
649	II	AEX30306	*Usnea longissima*	2141
650	V	AGI60157	*Usnea longissima*	1720
651	VI	AGI60156	*Usnea longissima*	2441
652	–	CCF48435	*Ustilago hordei*	1984
653	II	XP_003008898	*Verticillium alfalfae* VaMs.102	2005
654	II	EGY13508	*Verticillium dahliae* VdLs.17	2189
655	III	KDB16994	*Villosiclava virens*	2478
656	VII	KDB13139	*Villosiclava virens*	2641
657	VIII	AEF32750	*Volvariella volvacea*	2092
658	VII	ABS58604	*Xanthoparmelia semiviridis*	2164
659	II	AAM93545	*Xylaria* sp. BCC 1067	2162
660	II	XP_003848644	*Zymoseptoria tritici* IPO323	2175
661	VI	XP_003855816	*Zymoseptoria tritici* IPO323	2396

Table S2. List of 55 NR-PKSs related to known polyketides.

S/N	Group	Cyclization	Accession No.	Strain Name	Protein Name	Products	Ref.
1	I	C2-C7	XP_681178	*Aspergillus nidulans* FGSC A4	*A. nidulans* OrsA	Orsellinic acid	1
2	I	C2-C7	ACM42403	*Chaetomium chiversii*	*C. chiversii* RADS2	Radicicol	2
3	I	C2-C7	ABB90282	*Fusarium graminearum*	*F. graminearum* PKS13	Zearalenone	3
4	I	C2-C7	ACD39762	*Hypomyces subiculosus*	*H. subiculosus* Hpm3	Hypocemycin	4
5	I	C2-C7	ACD39770	*Metacordyceps chlamydosporia*	*M. chlamydosporia* RDC1	Radicicol	4
6	I	C3-C8	AGC95321	*Aspergillus terreus*	*A. terreus* AtCURS2	10,11-Dehydrocurvularin	5
7	II	C2-C7	BAD22832	*Bipolaris oryzae*	*B. oryzae* PKS1	THN	6,7
8	II	C2-C7	AAO60166	*Ceratocystis resinifera*	*C. resinifera* PKS1	THN	8,9
9	II	C2-C7	BAA18956	*Colletotrichum lagenaria*	*C. lagenarium* PKS1	THN	10,11
10	II	C2-C7	ABU63483	*Elsinoe fawcettii*	*E. fawcettii* PKS1	Elsinochrome	12,13
11	II	C2-C7	AAD31436	*Exophiala dermatitidis*	*E. dermatitidis* PKS1	THN	14
12	II	C2-C7	AAN75188	*Exophiala lecanii-corni*	*E. lecanii-corni* PKS1	THN	15
13	II	C2-C7	AAN59953	*Glarea lozoyensis*	*G. lozoyensis* PKS1	THN	16
14	II	C2-C7	AAD38786	*Nodulisporium* sp. ATCC74245	*Nodulisporium* sp. PKS1	THN	17
15	II	C2-C7	ABD47522	*Ophiostoma piceae*	*O. piceae* PKSA	THN	18
16	II	C2-C7	CAM35471	*Sordaria macrospora*	*S. macrospora* PKS	THN	19
17	III	C2-C7	AAC39471	*Aspergillus fumigatus*	*A. fumigatus* Alb1	Naphthopyrones	20,21
18	III	C2-C7	EDP55264	*Aspergillus fumigatus* A1163	*A. fumigatus* PksP	THN	22,23
19	III	C2-C7	Q03149	*Aspergillus nidulans* FGSC A4	*A. nidulans* WA	YWA1, Naphthopyrone	24
20	III	C2-C7	EHA28527	*Aspergillus niger* ATCC 1015	*A. niger* AlbA	YWA1, dimeric naphtho-γ-pyrones	25
21	III	C2-C7	CAB92399	*Fusarium fujikuroi*	*F. fujikuroi* PKS4	Bikaverin	26,27
22	III	C2-C7	AAU10633	*Fusarium graminearum*	*F. graminearum* PKS12	Aurofusarin	28,29

23	IV	C4-C9	AAA81586	*Aspergillus nidulans*	*A. nidulans* PKSST	sterigmatocystin	30
24	IV	C4-C9	Q12397	*Aspergillus nidulans* FGSC A4	*A. nidulans* StcA	sterigmatocystin	30
25	IV	C4-C9	ACH72912	*Aspergillus ochraceoroseus*	*A. ochraceoroseus* AflC	Aflatoxin	31
26	IV	C4-C9	BAE71314	*Aspergillus oryzae*	*A. oryzae* PKSA	Aflatoxin	32
27	IV	C4-C9	Q12053	*Aspergillus parasiticus*	*A. parasiticus* PksA	Aflatoxin	33
28	IV	C4-C9	AAT69682	*Cercospora nicotianae*	*C. nicotianae* CTB1	THN	34
29	IV	C4-C9	CCE67070	*Fusarium fujikuroi*	*F. fujikuroi* Fsr1	Fusarubin	35
30	IV	C4-C9	AAS92537	*Leptosphaeria maculans*	*L. maculans* PKS1	Sirodesmin PL	13
31	IV	C4-C9	AAZ95017	*Mycosphaerella pini*	*M. pini* PKSA	Aflatoxin	36
32	IV	C4-C9	XP_003039929	*Nectria haematococca* mpVI 77-13-4	*N. haematococca* PKS1	Bostrycoidin,fusarubin	37
33	V	C1-C6	ADI24953	*Penicillium aethiopicum*	*P. aethiopicum* GsfA	griseofulvin	38
34	V	C2-C7	XP_664675	*Aspergillus nidulans* FGSC A4	*A. nidulans* PkgA	dehydrocitreoisocoumarin, citreisocoumarin, alternariol	24
35	V	C6-C11	XP_746435	*Aspergillus fumigatus* Af293	*A. fumigatus* EncA	Endocrocin	39
36	V	C6-C11	XP_657754	*Aspergillus nidulans* FGSC A4	*A. nidulans* MdpG	Atrochrysone carboxylic acid	40
37	V	C6-C11	XP_663604	*Aspergillus nidulans* FGSC A4	*A. nidulans* AptA	Asperthecin	41
38	V	C6-C11	XP_001394705	*Aspergillus niger* CBS 513. 88	*A. niger* AdaA	TAN-1612, BMS-192548	42
39	V	C6-C11	XP_001217072	*Aspergillus terreus* NIH2624	*A. terreus* ACAS	Emodin	43
40	V	C6-C11	ADI24926	*Penicillium aethiopicum*	*P. aethiopicum* VrtA	viridicatumtoxin	38
41	VI	C2-C7	XP_681652	*Aspergillus nidulans* FGSC A4	*A. nidulans* AusA	3,5-dimethylorsellinic acid, austinol,dehydroaustinol	24
42	VI	C2-C7	ADY00130	*Penicillium brevicompactum*	*P. brevicompactum* MpaC	5-methylorsellinic acid, mycophenolic acid	44
43	VI	C2-C7	XP_664052	*Aspergillus nidulans* FGSC A4	*A. nidulans* PkbA	3-methylorsellinic acid,cichorine	24
44	VII	C2-C7	XP_658638	*Aspergillus nidulans* FGSC A4	*A. nidulans* AfoE	asperfuranone	45
45	VII	C2-C7	XP_660990	*Aspergillus nidulans* FGSC A4	*A. nidulans* PkiA	2,4-dihydroxy-3-methyl-6-(2-oxoundecyl)benzaldehyde	24
46	VII	C2-C7	XP_658127	*Aspergillus nidulans* FGSC A4	*A. nidulans* PkdA	2-ethyl-4,6-dihydroxy-3,5-dimethylbenzaldehyde	24

47	VII	C2-C7	XP_660834	*Aspergillus nidulans* FGSC A4	*A. nidulans* PkfA	orsellinaldehyde	24
48	VII	C2-C7	EHA28237	*Aspergillus niger* ATCC 1015	*A. nidulans* AzaA	Azanigerone A	46
49	VII	C2-C7	AGN71604	*Monascus pilosus*	*M. pilosus* PKS5	Rubropunctatin	47
50	VII	C2-C7	BAD44749	*Monascus purpureus*	*M. purpureus* PksCT	citrinin	48
51	VII	C2-C7	CAN87161	*Sarocladium strictum*	*S. strictum* MOS	3-methylorcinaldehyde	49
52	VII	C2-C7	XP_659636	*Aspergillus nidulans* FGSC A4	*A. nidulans* PkhA	2,4-dihydroxy-6-[(3E,5E,7E)-2-oxonona-3,5,7-trienyl]benzaldehyde	24
53	VII	C2-C7	XP_001212610	*Aspergillus terreus* NIH2624	*A. terreus* ATEG_03432	citrinin	43
54	VII	C2-C7	ANID_07903	*Aspergillus nidulans* FGSC A4	*A. nidulans* PkeA	2,4-dihydroxy-3-methyl-6-(2-oxopropyl)benzaldehyde	24
55	VIII	C2-C7	AFL91703	*Armillaria mellea*	*A. mellea* ArmB	Orsellinic acid	50

References

1 Schroeckh, V. *et al.* Intimate bacterial-fungal interaction triggers biosynthesis of archetypal polyketides in *Aspergillus nidulans*. *Proc Natl Acad Sci USA*. **106**, 14558-14563 (2009).

2 Wang, S. *et al.* Functional characterization of the biosynthesis of radicicol, an Hsp90 inhibitor resorcylic acid lactone from *Chaetomium chiversii*. *Chem Biol*. **15**, 1328-1338 (2008).

3 Gaffoor, I. & Trail, F. Characterization of two polyketide synthase genes involved in zearalenone biosynthesis in *Gibberella zeae*. *Appl Environ Microbiol*. **72**, 1793-1799 (2006).

4 Reeves, C. D., Hu, Z., Reid, R. & Kealey, J. T. Genes for the biosynthesis of the fungal polyketides hypothemycin from *Hypomyces subiculosus* and radicicol from *Pochonia chlamydosporia*. *Appl Environ Microbiol*. **74**, 5121-5129 (2008).

5 Xu, Y. *et al.* Characterization of the biosynthetic genes for 10,11-dehydrocurvularin, a heat shock response-modulating anticancer fungal polyketide from *Aspergillus terreus*. *Appl Environ Microbiol*. **79**, 2038-2047 (2013).

6 Moriwaki, A. *et al.* Insertional mutagenesis and characterization of a polyketide synthase gene (*PKS1*) required for melanin biosynthesis in *Bipolaris oryzae*. *FEMS Microbiol Lett*. **238**, 1-8 (2004).

7 Moriwaki, A., Ueno, M., Arase, S. & Kihara, J. RNA-mediated gene silencing in the phytopathogenic fungus *Bipolaris oryzae*. *FEMS Microbiol Lett*. **269**, 85-89 (2007).
8 Loppnau, P., Tanguay, P. & Breuil, C. Isolation and disruption of the melanin pathway polyketide synthase gene of the softwood deep stain fungus *Ceratocystis resinifera*. *Fungal Genetics and Biology*. **41**, 33-41 (2004).
9 Tanguay, P., Loppnau, P., Morin, C., Bernier, L. & Breuil, C. A spontaneous albino mutant of *Ceratocystis resinifera* results from a point mutation in the polyketide synthase gene, *PKS1*. *Can J Microbiol*. **52**, 501-507 (2006).
10 Takano, Y. *et al.* Structural analysis of *PKS1*, a polyketide synthase gene involved in melanin biosynthesis in *Colletotrichum lagenarium*. *Mol Gen Genet*. **249**, 162-167 (1995).
11 Fujii, I. *et al.* Enzymatic synthesis of 1,3,6,8-tetrahydroxynaphthalene solely from malonyl coenzyme A by a fungal iterative type I polyketide synthase *PKS1*. *Biochemistry*. **39**, 8853-8858 (2000).
12 Chung, K. R. & Liao, H. L. Determination of a transcriptional regulator-like gene involved in biosynthesis of elsinochrome phytotoxin by the citrus scab fungus, *Elsinoe fawcettii*. *Microbiology*. **154**, 3556-3566 (2008).
13 Liao, H. L. & Chung, K. R. Genetic dissection defines the roles of elsinochrome Phytotoxin for fungal pathogenesis and conidiation of the citrus pathogen *Elsinoe fawcettii*. *Mol Plant Microbe Interact*. **21**, 469-479 (2008).
14 Wheeler, M. H. *et al.* New biosynthetic step in the melanin pathway of *Wangiella* (*Exophiala*) *dermatitidis*: evidence for 2-acetyl-1,3,6,8-Tetrahydroxynaphthalene as a novel precursor. *Eukaryot Cell*. **7**, 1699-1711 (2008).
15 Cheng, Q., Kinney, K. A., Whitman, C. P. & Szaniszlo, P. J. Characterization of two polyketide synthase genes in *Exophiala lecanii-corni*, a melanized fungus with bioremediation potential. *Bioorg Chem*. **32**, 92-108 (2004).
16 Zhang, A. *et al.* Efficient disruption of a polyketide synthase gene (*pks1*) required for melanin synthesis through *Agrobacterium*-mediated transformation of *Glarea lozoyensis*. *Mol Genet Genomics*. **268**, 645-655 (2003).
17 Fulton, T. R., Ibrahim, N., Losada, M. C., Grzegorski, D. & Tkacz, J. S. A melanin polyketide synthase (PKS) gene from *Nodulisporium* sp. that shows homology to the *pks1* gene of *Colletotrichum lagenarium*. *Mol Gen Genet*. **262**, 714-720 (1999).
18 Tanguay, P., Tangen, K. & Breuil, C. Identifying Pigmentation-Related Genes in *Ophiostoma piceae* Using *Agrobacterium*-Mediated Integration. *Phytopathology*. **97**, 1040-1048 (2007).
19 Engh, I., Nowrousian, M. & Kuck, U. Regulation of melanin biosynthesis *via* the dihydroxynaphthalene pathway is dependent on sexual development in the ascomycete *Sordaria macrospora*. *FEMS Microbiol Lett*. **275**, 62-70 (2007).
20 Tsai, H. F., Chang, Y. C., Washburn, R. G., Wheeler, M. H. & Kwon-Chung, K. J. The developmentally regulated *alb1* gene of *Aspergillus fumigatus*: its role in

modulation of conidial morphology and virulence. *J Bacteriol*. **180**, 3031-3038 (1998).

21 Watanabe, A. *et al. Aspergillus fumigatus alb1* encodes naphthopyrone synthase when expressed in *Aspergillus oryzae*. *FEMS Microbiol Lett*. **192**, 39-44 (2000).

22 Langfelder, K. *et al.* Identification of a polyketide synthase gene (*pksP*) of *Aspergillus fumigatus* involved in conidial pigment biosynthesis and virulence. *Med Microbiol Immunol*. **187**, 79-89 (1998).

23 Fedorova, N. D. *et al.* Genomic islands in the pathogenic filamentous fungus *Aspergillus fumigatus*. *PLoS Genet*. **4**, e1000046 (2008).

24 Ahuja, M. *et al.* Illuminating the diversity of aromatic polyketide synthases in *Aspergillus nidulans*. *J Am Chem Soc*. **134**, 8212-8221 (2012).

25 Chiang, Y. M. *et al.* Characterization of a polyketide synthase in *Aspergillus niger* whose product is a precursor for both dihydroxynaphthalene (DHN) melanin and naphtho-gamma-pyrone. *Fungal Genet Biol*. **48**, 430-437 (2011).

26 Linnemannstons, P. *et al.* The polyketide synthase gene *pks4* from *Gibberella fujikuroi* encodes a key enzyme in the biosynthesis of the red pigment bikaverin. *Fungal Genet Biol*. **37**, 134-148 (2002).

27 Wiemann, P. *et al.* Biosynthesis of the red pigment bikaverin in *Fusarium fujikuroi*: genes, their function and regulation. *Mol Microbiol*. **72**, 931-946 (2009).

28 Frandsen, R. J. *et al.* Two novel classes of enzymes are required for the biosynthesis of aurofusarin in *Fusarium graminearum*. *J Biol Chem*. **286**, 10419-10428 (2011).

29 Maier, F. J., Malz, S., Losch, A. P., Lacour, T. & Schafer, W. Development of a highly efficient gene targeting system for *Fusarium graminearum* using the disruption of a polyketide synthase gene as a visible marker. *FEMS Yeast Res*. **5**, 653-662 (2005).

30 Yu, J. H. & Leonard, T. J. Sterigmatocystin biosynthesis in *Aspergillus nidulans* requires a novel type I polyketide synthase. *J Bacteriol*. **177**, 4792-4800 (1995).

31 Cary, J. W., Ehrlich, K. C., Beltz, S. B., Harris-Coward, P. & Klich, M. A. Characterization of the *Aspergillus ochraceoroseus* aflatoxin/sterigmatocystin biosynthetic gene cluster. *Mycologia*. **101**, 352-362 (2009).

32 Tominaga, M. *et al.* Molecular analysis of an inactive aflatoxin biosynthesis gene cluster in *Aspergillus oryzae* RIB strains. *Appl Environ Microbiol*. **72**, 484-490 (2006).

33 Feng, G. H. & Leonard, T. J. Characterization of the polyketide synthase gene (*pksL1*) required for aflatoxin biosynthesis in *Aspergillus parasiticus*. *J Bacteriol*. **177**, 6246-6254 (1995).

34 Chung, K. R., Ehrenshaft, M., Wetzel, D. K. & Daub, M. E. Cercosporin-deficient mutants by plasmid tagging in the asexual fungus *Cercospora nicotianae*. *Mol Genet Genomics*. **270**, 103-113 (2003).

35 Studt, L., Wiemann, P., Kleigrewe, K., Humpf, H. U. & Tudzynski, B. Biosynthesis of fusarubins accounts for pigmentation of *Fusarium fujikuroi* perithecia. *Appl Environ Microbiol*. **78**, 4468-4480 (2012).

36 Zhang, S., Schwelm, A., Jin, H., Collins, L. J. & Bradshaw, R. E. A fragmented aflatoxin-like gene cluster in the forest pathogen *Dothistroma septosporum*. *Fungal Genet Biol*. **44**, 1342-1354 (2007).

37 Awakawa, T., Kaji, T., Wakimoto, T. & Abe, I. A heptaketide naphthaldehyde produced by a polyketide synthase from *Nectria haematococca*. *Bioorg Med Chem Lett*. **22**, 4338-4340 (2012).

38 Chooi, Y. H., Cacho, R. & Tang, Y. Identification of the viridicatumtoxin and griseofulvin gene clusters from *Penicillium aethiopicum*. *Chem Biol*. **17**, 483-494 (2010).

39 Lim, F. Y. *et al.* Genome-Based Cluster Deletion Reveals an Endocrocin Biosynthetic Pathway in *Aspergillus fumigatus*. *Applied and Environmental Microbiology*. **78**, 6395-6395 (2012).

40 Chiang, Y. M. *et al.* Characterization of the *Aspergillus nidulans* Monodictyphenone Gene Cluster. *Applied and Environmental Microbiology*. **76**, 2067-2074 (2010).

41 Szewczyk, E. *et al.* Identification and characterization of the asperthecin gene cluster of *Aspergillus nidulans*. *Appl Environ Microbiol*. **74**, 7607-7612 (2008).

42 Li, Y., Chooi, Y. H., Sheng, Y., Valentine, J. S. & Tang, Y. Comparative characterization of fungal anthracenone and naphthacenedione biosynthetic pathways reveals an alpha-hydroxylation-dependent Claisen-like cyclization catalyzed by a dimanganese thioesterase. *J Am Chem Soc*. **133**, 15773-15785 (2011).

43 Boruta, T. & Bizukojc, M. Culture-based and sequence-based insights into biosynthesis of secondary metabolites by *Aspergillus terreus* ATCC 20542. *J Biotechnol*. **175**, 53-62 (2014).

44 Regueira, T. B. *et al.* Molecular basis for mycophenolic acid biosynthesis in *Penicillium brevicompactum*. *Appl Environ Microbiol*. **77**, 3035-3043 (2011).

45 Chiang, Y. M. *et al.* A gene cluster containing two fungal polyketide synthases encodes the biosynthetic pathway for a polyketide, asperfuranone, in *Aspergillus nidulans*. *J Am Chem Soc*. **131**, 2965-2970 (2009).

46 Zabala, A. O., Xu, W., Chooi, Y. H. & Tang, Y. Characterization of a silent azaphilone gene cluster from *Aspergillus niger* ATCC 1015 reveals a hydroxylation-mediated pyran-ring formation. *Chem Biol*. **19**, 1049-1059 (2012).

47 Balakrishnan, B. *et al.* Genetic localization and *in vivo* characterization of a *Monascus azaphilone* pigment biosynthetic gene cluster. *Appl Microbiol Biotechnol*. **97**, 6337-6345 (2013).

48 Shimizu, T. *et al.* Polyketide synthase gene responsible for citrinin biosynthesis in *Monascus purpureus*. *Appl Environ Microbiol*. **71**, 3453-3457 (2005).

49 Bailey, A. M. *et al.* Characterisation of 3-methylorcinaldehyde synthase (MOS) in *Acremonium strictum*: first observation of a reductive release mechanism during polyketide biosynthesis. *Chem Commun (Camb)*, 4053-4055 (2007).

50 Lackner, G., Bohnert, M., Wick, J. & Hoffmeister, D. Assembly of melleolide antibiotics involves a polyketide synthase with cross-coupling activity. *Chem Biol*. **20**, 1101-1106 (2013).

Table S3. Structural prediction of PT domains.

Group	Accession No.	Length	Iden	Cov	Z-score	Method	TM-score	Ramachandran plot (%)			
								core	allow	gener	disall
I	ABB90282	339	0.16	0.92	2.40	A	0.95	79.1	17.7	1.4	1.8
						B	0.96	74.8	20.6	2.5	2.1
						C	0.96	76.4	21.1	1.8	0.7
I	ACD39762	342	0.19	0.91	2.47	A	0.95	76.4	19.2	3.4	1.0
						B	0.96	71.0	25.9	2.0	1.0
						C	0.95	76.4	18.8	3.1	1.7
I	AGC95321	354	0.21	0.88	2.37	A	0.94	73.7	22.1	2.8	1.4
						B	0.95	68.0	24.2	4.6	3.2
						C	0.95	77.0	19.1	1.4	2.5
I	XP_681178	339	0.21	0.92	2.52	A	0.94	73.9	22.0	2.1	2.1
						B	0.95	74.2	20.6	3.8	1.4
						C	0.95	75.8	20.1	2.8	1.4
II	AAN75188	345	0.25	0.90	2.46	A	0.96	73.1	24.0	1.8	1.1
						B	0.96	73.2	21.8	2.9	2.1
						C	0.96	74.3	22.5	2.9	0.4
II	ABU63483	358	0.24	0.88	2.45	A	0.95	74.4	21.8	2.5	1.4
						B	0.95	70.4	21.6	3.5	4.5
						C	0.96	77.5	18.9	2.1	1.4
II	BAA18956	342	0.32	0.91	2.57	A	0.96	76.7	17.7	3.2	2.5
						B	0.96	76.8	18.7	3.5	1.1
						C	0.96	75.4	19.9	3.6	1.1
III	AAU10633	344	0.26	0.91	2.40	A	0.93	74.7	20.6	3.2	1.4
						B	0.95	69.0	21.7	6.0	3.2
						C	0.96	77.1	18.6	2.1	2.1
III	EDP55264	348	0.31	0.89	2.40	A	0.96	74.7	23.2	1.1	1.1
						B	0.96	77.2	18.2	3.9	0.7
						C	0.96	78.8	19.1	1.4	0.7
III	Q03149	341	0.30	0.91	2.42	A	0.95	79.2	17.6	2.8	0.4
						B	0.96	73.9	22.0	2.8	1.4
						C	0.96	75.2	22.0	2.1	0.7
IV	CCE67070	352	0.40	0.89	2.49	A	0.94	72.5	22.6	3.1	1.7
						B	0.97	75.4	19.7	2.4	2.4
						C	0.96	81.8	15.4	1.1	1.8
IV	Q12397	369	0.72	0.86	2.52	A	0.96	76.5	20.8	2.0	0.7
						B	0.96	76.5	18.4	2.4	2.7
						C	0.96	79.8	18.2	1.0	1.0

V	ADI24953	384	0.20	0.81	2.25	A	0.94	73.9	20.5	2.5	3.2
						B	0.94	69.0	22.2	5.6	3.2
						C	0.94	74.6	22.1	2.1	1.1
V	XP_001394705	379	0.24	0.83	2.37	A	0.93	75.8	18.3	2.4	3.5
						B	0.94	71.8	22.3	4.8	1.0
						C	0.94	85.6	12.6	1.1	0.7
V	XP_657754	366	0.25	0.86	2.37	A	0.95	72.5	23.2	2.5	1.8
						B	0.95	68.4	25.6	2.5	3.5
						C	0.95	78.0	18.8	2.5	0.7
V	XP_663604	372	0.22	0.84	2.37	A	0.93	72.9	21.9	3.1	2.1
						B	0.95	73.1	20.0	4.5	2.4
						C	0.96	72.5	24.7	1.7	1.0
VI	ADY00130	359	0.17	0.86	2.48	A	0.92	76.5	17.6	3.7	2.2
						B	0.92	67.9	25.1	3.1	3.8
						C	0.94	76.6	19.1	2.5	1.8
VI	XP_664052	367	0.18	0.84	3.02	A	0.93	72.8	22.8	2.6	1.8
						B	0.92	69.2	24.7	3.8	2.4
						C	0.93	75.5	20.0	2.4	2.1
VI	XP_681652	355	0.17	0.86	2.17	A	0.92	79.5	16.9	2.2	1.4
						B	0.93	69.5	23.5	4.9	2.1
						C	0.94	76.4	20.7	1.8	1.1
VII	XP_001212610	368	0.15	0.83	2.02	A	0.92	74.5	22.6	2.2	0.7
						B	0.93	68.8	22.6	5.2	3.5
						C	0.92	76.1	20.4	2.1	1.4
VII	XP_658638	372	0.17	0.83	2.08	A	0.90	70.6	23.3	3.6	2.5
						B	0.92	65.9	29.0	2.4	2.7
						C	0.91	69.8	27.1	1.7	1.4
VII	XP_660834	351	0.13	0.87	2.02	A	0.89	65.4	28.2	4.3	2.1
						B	0.90	69.1	22.0	5.5	3.4
						C	0.89	72.2	23.1	3.6	1.1
VIII	AFL91703	367	0.21	0.83	3.31	A	0.93	75.1	20.8	3.8	0.3
						B	0.93	70.7	24.4	3.1	1.7
						C	0.94	75.4	19.2	3.9	1.4

Ident is the percentage sequence identity between query sequence and the 3HRR sequence.

Cov represents the coverage of the modeling alignment and is equal to the number of aligned residues divided by the length of query protein.

Z-score is the normalized Z-score of the modeling alignments. Alignment with a normalized Z-score >1 mean a good alignment and vice versa.

Method: A - SWISS-MODEL+MD, B - I-TASSER+MD, C - PHYRE2+MD.

Ramachandran plot: core - residues in most favoured regions, allow - residues in additional allowed regions, gener - residues in generously allowed regions, disall - residues in disallowed regions.

Table S4. Information of PT domains.

Group	Cyclization	Accession No.	Cavity Volume (Å^3)	Number of CLRs
I	C2-C7	XP_681178	927.5	37
I	C2-C7	ABB90282	776.6	31
I	C2-C7	ACD39762	899.5	32
I	C3-C8	AGC95321	672.2	30
II	C2-C7	BAA18956	975.5	36
II	C2-C7	AAN75188	894.4	32
II	C2-C7	ABU63483	1097.3	38
III	C2-C7	Q03149	953.8	37
III	C2-C7	AAU10633	1040.0	38
III	C2-C7	EDP55264	1049.3	38
IV	C4-C9	Q12053 (3HRR)	1358.9	45
IV	C4-C9	Q12397	1264.9	43
IV	C4-C9	CCE67070	1019.1	36
V	C6-C11	XP_657754	1181.3	41
V	C6-C11	XP_663604	1116.4	47
V	C6-C11	XP_001394705	1443.2	50
V	C1-C6	ADI24953	634.3	31
VI	C2-C7	XP_681652	938.6	34
VI	C2-C7	ADY00130	887.0	32
VI	C2-C7	XP_664052	968.5	32
VII	C2-C7	XP_658638	1187.0	43
VII	C2-C7	XP_001212610	954.3	44
VII	C2-C7	XP_660834	876.6	35
VIII	C2-C7	AFL91703	773.9	29

Table S5. Conservation analysis of CLRs in PT domains.

I			II			III			IV			V			VI			VII			VIII		ALL
SEQ	G	P	SEQ	G	P	SEQ	G	P	SEQ	G	P	SEQ	G	P	SEQ	G	P	SEQ	G	P	SEQ	G	G
H1304	9	Y	H1330	9	Y	H1326	9	Y	H1345	9	Y	H1376	9	Y	H1310	9	Y	H1415	9	Y	H1338	9	9
V1306	8	Y	C1332	8	Y	V1328	9	Y	V1347	9	Y	M1378	9	Y	V1312	9	Y	I1417	7	Y	V1340	9	9
I1311	8	Y	L1337	9	Y	L1333	8	Y	L1352	9	Y	V1383	9	Y	L1317	8	Y	I1423	8	Y	L1345	9	9
C1312	7	Y	C1338	7	Y	C1334	7	Y	C1353	6	Y	V1384	8	Y	C1318	8	Y	C1424	7	Y	C1346	9	8
P1313	9	Y	P1339	9	Y	P1335	8	Y	T1354	9	Y	T1385	9	Y	P1319	9	Y	P1425	9	Y	P1347	9	9
A1314	8	Y	S1340	9	Y	S1336	9	Y	P1355	9	Y	S1386	9	Y	A1320	9	Y	G1426	8	Y	A1348	9	9
S1315	8		T1341	8	Y	S1337	9	Y	S1356	9		S1387	9	Y	S1321	9		T1427	8	Y	S1349	9	9
I1316	8		L1342	7		L1338	7		F1357	8		I1388	9		L1322	7		L1428	6	Y	V1350	9	8
F1317	8		Y1343	8	Y	Y1339	8	Y	Y1358	9	Y	H1389	9		Y1323	9	Y	E1429	7	Y	Y1351	9	8
I1318	4		A1344	6		A1340	8		A1359	9		G1390	7	Y	F1324	7		C1430	6		H1352	4	7
L1346	5		V1372	9		V1368	7		M1392	8		L1418	7	Y	L1349	8		M1456	7		I1380	8	8
M1348	7	Y	V1374	7	Y	V1370	7	Y	V1394	7	Y	V1420	9	Y	I1351	8	Y	N1458	8	Y	Y1382	7	8
H1349	3	Y	P1375	3	Y	P1371	4	Y	D1395	6	Y	L1421	4	Y	C1352	5	Y	H1459	6	Y	V1383	4	4
S1350	8	Y	K1376	8	Y	K1372	7	Y	K1396	8	Y	R1422	7	Y	S1353	8	Y	S1460	6	Y	K1384	4	7
P1351	8	Y	T1377	6	Y	P1373	7	Y	A1397	9	Y	G1423	8	Y	P1354	8	Y	P1461	8	Y	P1385	9	8
L1352	7	Y	L1378	7	Y	L1374	9	Y	L1398	9	Y	L1424	8		L1355	9	Y	I1462	7	Y	L1386	9	8
V1353	8		I1379	8		I1375	7		V1399	8	Y	V1425	8		V1356	8		C1463	7		V1387	9	8

V1390	2		T1415	4		I1411	2		T1442	7	Y	E1465	5		–	–		E1501	6		P1420	4	4
Y1392	5		H1417	9		H1413	9		H1444	9	Y	F1467	7	Y	H1393	9		H1503	9	Y	S1422	5	8
G1393	9		A1418	8		A1414	9		A1445	8		A1468	8		A1394	9		A1504	8	Y	V1423	5	8
M1436	7		I1460	6		L1456	4		Y1486	6	Y	F1510	5		L1437	7		L1553	8		I1466	7	6
D1437	5		G1461	4		R1457	7		N1487	8	Y	T1511	8		K1438	6		Q1554	8		T1467	7	7
T1438	6		R1462	9		R1458	8		R1488	5	Y	R1512	4		R1439	8		G1555	9		T1468	7	7
F1441	8		T1465	9		V1461	7		G1491	6	Y	A1515	8		V1442	8		V1558	8		A1471	7	8
Y1442	9		Y1466	9		Y1462	9		Y1492	9	Y	Y1516	9		Y1443	9		Y1559	9		Y1472	9	9
F1445	9	Y	F1469	9	Y	F1465	9	Y	M1495	8	Y	F1519	9	Y	F1446	8	Y	F1562	8	Y	F1476	9	9
I1448	7	Y	L1472	7	Y	L1468	5	Y	M1498	7	Y	L1523	8	Y	A1449	8	Y	I1565	7	Y	V1479	9	8
V1449	9	Y	V1473	9	Y	V1469	9	Y	A1499	9	Y	V1524	9	Y	V1450	9	Y	V1566	9	Y	V1480	9	9
Y1451	9	Y	Y1475	9	Y	Y1471	9	Y	F1501	9	Y	Y1526	9	Y	Y1452	9	Y	Y1568	9	Y	Y1482	9	9
V1458	6		M1482	9		I1478	8		L1508	8	Y	M1533	9		V1459	9		V1575	7		M1489	7	8
E1460	8		E1484	7		E1480	8		Y1510	5	Y	S1535	8		E1461	6		Y1577	7		N1491	5	7
A1461	8		V1485	9		V1481	8		L1511	5	Y	V1536	9		V1462	8		V1578	7		V1492	7	8
V1492	5		I1516	9		I1510	7	Y	V1542	8		I1564	9	Y	I1490	6		S1606	7		M1523	7	7
D1493	9	Y	D1517	9	Y	D1511	9	Y	D1543	9	Y	D1565	9	Y	D1491	9	Y	D1607	9	Y	D1524	9	9
L1495	6		C1519	3		F1513	7		I1545	5		V1567	8	Y	F1493	9		F1609	8		M1526	8	7
V1496	7	Y	C1520	5	Y	G1514	9	Y	T1546	8	Y	A1568	7	Y	I1494	7	Y	S1610	7	Y	L1527	9	7
H1497	9	Y	H1521	9	Y	H1515	8	Y	Q1547	9	Y	H1569	9	Y	Q1495	9	Y	Q1611	9	Y	H1528	9	9
G1500	9	Y	G1524	9	Y	G1518	9	Y	G1550	8	Y	G1572	9	Y	G1498	9	Y	G1614	9	Y	G1531	9	9

F1501	9		F1525	8		F1519	9		F1551	9	Y	F1573	8		I1499	8	Y	V1615	8	Y	F1532	9	9
N1504	9	Y	N1528	9	Y	N1522	9	Y	N1554	9	Y	N1576	9	Y	N1502	9	Y	N1618	9	Y	N1535	9	9
V1514	5	Y	V1539	9		V1533	9	Y	V1565	8		Y1587	8	Y	V1512	8	Y	M1628	7	Y	A1543	8	8
H1515	6	Y	F1540	5	Y	F1534	6	Y	Y1566	6	Y	C1588	6	Y	F1513	6	Y	F1629	6	Y	Y1544	7	6
I1516	8	Y	I1541	8	Y	V1535	8	Y	V1567	8	Y	V1589	9	Y	V1514	8	Y	I1630	8	Y	I1545	9	8
A1517	7	Y	S1542	9	Y	N1536	9	Y	N1568	9	Y	T1590	9	Y	C1515	8	Y	A1631	8	Y	C1546	9	9
N1518	5	Y	H1543	9	Y	H1537	9	Y	H1569	9	Y	P1591	8	Y	S1516	8	Y	T1632	6	Y	S1547	6	8
H1519	8		G1544	9		G1538	9		G1570	9		G1592	9		S1517	5		G1633	7	Y	K1548	4	7
I1520	6		W1545	9	Y	W1539	9	Y	W1571	9		W1593	6	Y	V1518	7		C1634	6	Y	V1549	8	8
L1523	3		M1548	5		M1542	7		F1574	6		L1596	6	Y	V1521	6		S1637	6	Y	V1552	7	6
I1525	5		F1550	7		C1544	4		I1576	6		F1598	6	Y	I1523	4		M1638	6		A1554	5	5
L1529	7		L1554	6		F1548	5		L1580	6		L1602	8	Y	F1527	7		–	–		I1559	4	5
V1555	8		A1579	9		V1572	9		V1605	7		V1628	9	Y	V1556	8		L1670	7		A1584	8	8
T1557	8		V1581	7		I1574	6		V1607	7		I1630	7	Y	V1558	9		V1672	7		A1586	9	8
A1566	7		G1589	7		A1582	8		A1615	8		G1638	9	Y	V1568	5		E1682	9		A1597	9	8
C1568	5		A1591	3		F1584	2	Y	F1617	7		C1640	7	Y	M1570	7		M1684	8		L1599	6	6
I1571	7		V1594	5	Y	V1587	8	Y	L1620	8		I1643	8	Y	A1573	8		V1687	7	Y	M1602	8	8
C1572	3		R1595	7		K1588	6		T1621	6		Q1644	6		T1574	5		Q1688	6	Y	C1603	4	5
F1573	9	Y	F1596	9	Y	F1589	8	Y	L1622	6	Y	F1645	9	Y	F1575	9	Y	Y1689	8	Y	F1604	9	9
K1574	7		A1597	8	Y	Q1590	5	Y	R1623	9	Y	R1646	8	Y	T1576	6	Y	M1690	5		K1605	7	7
L1576	7	Y	I1599	8	Y	L1592	7	Y	V1625	9	Y	Y1648	8	Y	V1578	7	Y	V1692	8	Y	L1607	7	8

F1580	4	Y	V1603	8		I1596	6		A1629	6		L1652	9		S1582	8		S1696	7		T1611	6	7
F1581	7	Y	L1604	7	Y	L1597	8	Y	L1630	8	Y	L1653	9	Y	L1583	9	Y	M1697	8	Y	L1612	7	8
L1584	4	Y	V1607	4	Y	A1600	6		V1633	8	Y	F1656	8	Y	T1586	5	Y	M1700	4	Y	S1615	6	6
T1585	7	Y	L1608	8	Y	L1601	7	Y	L1634	8	Y	F1657	9	Y	L1587	9	Y	L1701	8	Y	L1616	8	9
A1587	2		–	–		–	–		T1636	4	Y	A1659	7		R1589	6		R1703	6		M1618	4	5

SEQ: Single-letter abbreviations of amino acids and their position number. Group I prevail in XP_681178, group II prevail in BAA18956, group III prevail in Q03149, group IV prevail in Q12053 (3HRR), group V prevail in XP_657754, group VI prevail in XP_681652, group VII prevail in XP_658638, group VIII prevail in AFL91703.

G: Evolutionary conservation scores of the residues, analyzed by ConSurf (9, conserved; 1, variable).

P: Y, the residue is a CLR.

■: The red block, 27 common CLR sites in seven groups. ■:The green block, 11 unique CLR sites in group IV. ■:The yellow block,8 unique CLR sites in group V.
